# Tissue engineered vascular grafts transform into autologous neovessels capable of native function and growth

**DOI:** 10.1038/s43856-021-00063-7

**Published:** 2022-01-10

**Authors:** Kevin M. Blum, Jacob C. Zbinden, Abhay B. Ramachandra, Stephanie E. Lindsey, Jason M. Szafron, James W. Reinhardt, Megan Heitkemper, Cameron A. Best, Gabriel J. M. Mirhaidari, Yu-Chun Chang, Anudari Ulziibayar, John Kelly, Kejal V. Shah, Joseph D. Drews, Jason Zakko, Shinka Miyamoto, Yuichi Matsuzaki, Ryuma Iwaki, Hira Ahmad, Robbie Daulton, Drew Musgrave, Matthew G. Wiet, Eric Heuer, Emily Lawson, Erica Schwarz, Michael R. McDermott, Rajesh Krishnamurthy, Ramkumar Krishnamurthy, Kan Hor, Aimee K. Armstrong, Brian A. Boe, Darren P. Berman, Aaron J. Trask, Jay D. Humphrey, Alison L. Marsden, Toshiharu Shinoka, Christopher K. Breuer

**Affiliations:** 1 Center for Regenerative Medicine, Abigail Wexner Research Institute at Nationwide Children’s Hospital, Columbus, OH 43205 USA; 2 Department of Biomedical Engineering, The Ohio State University, Columbus, OH 43210 USA; 3 Department of Biomedical Engineering, Yale University, New Haven, CT 06520 USA; 4 Department of Pediatrics (Cardiology), Stanford University, Stanford, CA 94305 USA; 5 Institute for Computational and Mathematical Engineering (ICME), Stanford University, Stanford, CA 94305 USA; 6 The Ohio State University College of Medicine, Columbus, OH 43210 USA; 7 The Heart Center, Nationwide Children’s Hospital, Columbus, OH 43205 USA; 8 Department of Surgery, The Ohio State University Wexner Medical Center, Columbus, OH 43210 USA; 9 Department of Cardiovascular Surgery at Tokyo Women’s Medical University, Tokyo, Japan; 10 Department of Pediatric Colorectal and Pelvic Reconstructive Surgery, Nationwide Children’s Hospital, Columbus, OH 43205 USA; 11 University of Cincinnati College of Medicine 3230 Eden Ave, Cincinnati, OH 45267 USA; 12 Department of Bioengineering, Stanford University, Stanford, CA 94304 USA; 13 Center for Cardiovascular Research, Abigail Wexner Research Institute at Nationwide Children’s Hospital, Columbus, OH 43205 USA; 14 Department of Radiology, Nationwide Children’s Hospital, Columbus, Ohio 43205 USA; 15 Department of Pediatrics, The Ohio State University College of Medicine, Columbus, OH 43210 USA; 16 Department of Cardiothoracic Surgery, The Ohio State University College of Medicine, Columbus, OH 43205 USA

**Keywords:** Biomaterials, Tissues, Cardiovascular biology

## Abstract

**Background:**

Tissue-engineered vascular grafts (TEVGs) have the potential to advance the surgical management of infants and children requiring congenital heart surgery by creating functional vascular conduits with growth capacity.

**Methods:**

Herein, we used an integrative computational-experimental approach to elucidate the natural history of neovessel formation in a large animal preclinical model; combining an in vitro accelerated degradation study with mechanical testing, large animal implantation studies with in vivo imaging and histology, and data-informed computational growth and remodeling models.

**Results:**

Our findings demonstrate that the structural integrity of the polymeric scaffold is lost over the first 26 weeks in vivo, while polymeric fragments persist for up to 52 weeks. Our models predict that early neotissue accumulation is driven primarily by inflammatory processes in response to the implanted polymeric scaffold, but that turnover becomes progressively mechano-mediated as the scaffold degrades. Using a lamb model, we confirm that early neotissue formation results primarily from the foreign body reaction induced by the scaffold, resulting in an early period of dynamic remodeling characterized by transient TEVG narrowing. As the scaffold degrades, mechano-mediated neotissue remodeling becomes dominant around 26 weeks. After the scaffold degrades completely, the resulting neovessel undergoes growth and remodeling that mimicks native vessel behavior, including biological growth capacity, further supported by fluid–structure interaction simulations providing detailed hemodynamic and wall stress information.

**Conclusions:**

These findings provide insights into TEVG remodeling, and have important implications for clinical use and future development of TEVGs for children with congenital heart disease.

## Abstract (web summary)

Blum et al. combine computational and experimental methods to study the long-term development of tissue engineered vascular grafts in a lamb model. The authors demonstrate that the grafts undergo growth and remodeling, evolving to mimic the characteristics and function of a native blood vessel.

## Plain language summary

Surgery to correct defects in the heart that are present at birth sometimes requires the use of artificial blood vessels called vascular grafts. Tissue-engineered vascular grafts (TEVGs) are scaffolds seeded with cells that can develop into functional blood vessels over time. We conducted a series of laboratory and computer-based experiments to investigate how TEVGs develop into functional blood vessels, and demonstrated two phases of changes to the TEVG after implantation: an early phase driven by inflammation, and a later phase driven by the mechanical properties of the tissue. At later time points, the resulting blood vessels demonstrated the ability to grow and respond to blood flow in similar ways to the body’s own blood vessels. These results provide insight into the processes by which TEVGs become functional blood vessels, with implications for future clinical use of this technology.

## Introduction

The development of a vascular conduit with growth potential holds great promise for advancing the field of congenital heart surgery, where the process of normal growth may result in the patient out-growing a synthetic graft and requiring additional surgery^1,2^. A promising solution to this problem is the use of tissue engineering methods to create a living vascular conduit that can grow with the patient, thus mitigating risks associated with somatic overgrowth^3^. Several different groups have developed tissue-engineered vascular grafts (TEVGs) specifically for congenital heart surgery^4–10^. Results of preclinical and clinical studies have confirmed the ability of TEVGs to form living vascular conduits including demonstration of biological growth potential in some TEVGs^5,11–14^.

Multiple teams have advanced the clinical translation of TEVGs for use in the clinic; however, there are currently no TEVGs approved for use in the United States. To date, most of the translational work has been performed on small-diameter TEVGs designed for arterial bypass or arteriovenous angioaccess in adults^15–21^. These TEVGs are made using different materials and fabrication methods and typically suffer from different complications (aneurismal dilation or thrombosis rather than stenosis) than our large-diameter TEVGs designed for use in the Fontan circulation. The TEVG most comparable to ours is the unseeded, bioabsorbable TEVG manufactured by Xeltis, which has been implanted as an extracardiac conduit in 5 patients undergoing modified Fontan procedures in Russia. Results of this pilot study revealed no graft-related complications within 2 years after implantation^5,22,23^. The early results of this ongoing study are promising. Further follow-up is needed to fully evaluate the growth potential of the Xeltis TEVG in children^5,23^.

We previously developed a TEVG designed specifically for use in children with congenital heart disease who require surgical implantation of a vascular conduit^13^. This TEVG was made by seeding autologous bone marrow-derived cells onto a biodegradable tubular scaffold. Once seeded, the scaffold was implanted as a vascular conduit, with neotissue forming as the scaffold degraded in vivo. The TEVG was designed for use in children with single ventricle heart disease undergoing a Fontan operation, in which a vascular conduit is used to connect the inferior vena cava (IVC) to the right pulmonary artery^24^. Results of our first FDA-approved clinical trial evaluating this TEVG demonstrated an early period of dynamic remodeling ultimately resulting in the development of critical TEVG stenosis (>50% narrowing) in 3 of the first 4 patients within 6 months of implantation. All patients who developed critical stenosis were successfully treated with balloon angioplasty and have had no additional graft-related complications more than 5 years after implantation^11,12,25^. Due to this unanticipated graft-related complication, however, we closed the clinical study.

To improve our understanding of the processes underlying the formation of TEVG stenosis, we developed a computational model of growth and remodeling (G&R), which accurately predicted the formation of TEVG stenosis experienced in our clinical trial^11,26^. This model built upon more than a decade of experience in modeling G&R for native vessels, first in response to changing mechanobiological stimuli, then immunobiological stimuli^27–30^. An underlying concept in native vessel G&R is that they tend to exhibit mechanobiological homeostasis; that is, vascular cells may alter gene expression to return key mechano-regulated variables towards a homeostatic set-point^29,31^. Both flow-induced wall shear stress and pressure-induced intramural stress both serve as useful mechano-regulated variables that can be directly measured, analytically approximated, or computed using computational biomechanics. Simplified, a wall shear stress set-point tends to regulate luminal diameter while an intramural stress set-point tends to regulate wall thickness. Both of these processes are absent or dysregulated during the development of stenosis, suggesting loss of mechanical homeostasis and/or additional stimuli. The computational G&R model suggested that the clinically-observed TEVG stenosis arose from an inflammation-driven, mechano-mediated process with the unexpected prediction that such a stenosis should spontaneously reverse over time with a resolution of the inflammatory response. We validated this model-generated hypothesis in a lamb study that confirmed the reversible nature of our TEVG’s stenosis^11^.

Interestingly, since the TEVG computational G&R model was based on a model originally developed to describe and predict native vessel behavior, but modified to account for the biodegradable scaffold and its associated response^32^, it hypothesizes that upon complete scaffold degradation, our TEVG should transform into a neovessel that mimics the behavior of a native blood vessel. Herein, we tested this additional computational model-based hypothesis and quantified G&R and hemodynamics in the TEVG using a computational-experimental approach to better understand the natural history of neovessel formation as it applies to the clinical use of the TEVG.

## Methods

### Computational modeling: tissue engineered vascular graft growth and remodeling

G&R of TEVGs was simulated using a constrained mixture framework outlined in detail previously^11^. Briefly, the mass density of each structurally significant wall constituent, including polymer, smooth muscle cells, and collagen fibers, was tracked over time via a series of quasi-equilibrated steps. The mass densities of these constituents were separated into those produced via mechanobiological processes and those produced via immunological processes with:

1 $${\rho }^{{{{{{\rm{mech}}}}}}}(s)={\int }_{0}^{s}{m}_{h}^{{{{{{\rm{mech}}}}}}}(1+{\Upsilon }^{{{{{{\rm{mech}}}}}}}(\tau ))(1-\exp (-\tau )){q}^{{{{{{\rm{mech}}}}}}}(s,\tau ){{{{{\rm{d}}}}}}\tau$$

and

2 $${\rho }^{{{{{{\rm{infl}}}}}}}(s)={\int }_{0}^{s}{m}_{h}^{{{{{{\rm{infl}}}}}}}({\Upsilon }^{{{{{{\rm{infl}}}}}}}(\tau ))(1-\exp (-\tau )){q}^{{{{{{\rm{infl}}}}}}}(s,\tau ){{{{{\rm{d}}}}}}\tau$$

respectively. For each constituent type, ${m}_{h}^{{{{{{\rm{infl}}}}}}}$ and ${m}_{h}^{{{{{{\rm{mech}}}}}}}$ are the basal (homeostatic) production rates, ${\Upsilon }^{{{{{{\rm{infl}}}}}}}(\tau )$ and ${\Upsilon }^{{{{{{\rm{mech}}}}}}}(\tau )$ are the time-dependent gain functions, and *q*^infl^ and *q*^mech^ track the survival fraction of material produced at past time *τ* that remains at current time *s*. Specific functional forms and parameter values for the gain functions and survival functions were the same as those used previously, and the solution code and detailed methods, including the theoretical background were uploaded supplementally^11^. Constituent deformations were tracked from their constituent-specific stress-free natural configurations into the current equilibrated state via a multiplicative combination of deformations. Mechanical equilibrium was ensured at each time step to identify the evolving loaded geometry of the TEVG.

Case studies were performed that modified the kinetics for both the immuno- and mechano-mediated constituents. We simulated 4 cases: a case wherein we fit evolving IVUS data for implanted TEVGs^11^, a case with the immuno-mediated production set to zero ${\Upsilon }^{{{{{{\rm{infl}}}}}}}(\tau )=0$, and a case where the mechano-mediated constituents were not considered ${\rho }^{{{{{{\rm{mech}}}}}}}(s)=0$, and a case where mechano-mediated attenuation of native-like neotissue production was removed ${\Upsilon }^{{{{{{\rm{mech}}}}}}}(\tau )=0$.

### Sheep implantation and follow-up

#### Study design

All animal studies were performed according to ARRIVE Guidelines and under the approval and guidance of the NCH Animal Welfare and Resource Committee (Approval AR13-00079). The objective of this study was to quantify the natural history of neotissue formation and neovessel development in an established IVC interposition TEVG model, as sheep have been shown to be an ideal large animal model to compare to the physiology, hemodynamics, and growth capacity of human patients^10,33,34^. Sheep are housed in facilities that are USDA licensed and AAALAC accredited. Housing space for sheep is in accordance with the 2011 Guide for the Care and Use of Laboratory Animals (including HVAC parameters, lighting, and airflow). Sheep are housed in stainless steel runs with vinyl-coated and/or fiberglass flooring or in open flooring rooms with bedding on the floors. Water is provided ad libitum via an automatic watering system, and sheep receive nutritionally complete grain and a mixture of alfalfa and orchard grass hay placed into appropriate feeder devices daily. A complete mineral supplement is also provided ad libitum. Enrichment for sheep includes daily nutritious food items, social housing, positive daily interaction with husbandry staff, access to species specific items for direct manipulation (hanging toys for chewing or making noise), music for sensory stimulation and exercise outside of their primary enclosures on a bi-weekly basis. Seeded TEVGs were implanted in 53 lambs, as determined from previous experiments and through FDA evaluations, both male and females aged 4–8 months at implantation, and in vivo data were collected via serial angiography and intravascular ultrasound at 1, 6, 26, 52, 104, and 156 weeks^34^. A full list of included animals and inclusion in each protocol is available in Supplemental Data 2. The 1-week time point was used for baseline anatomic information, providing comparable data to the immediate post-operative period, while allowing the animal to recover from the initial surgical insult and decreasing risks associated with a prolonged anesthesia that would be needed to perform the implantation surgery and an initial catheterization during the same period. The primary endpoint was the narrowest cross-sectional area of the graft on IVUS imaging at each time. No data were excluded from the angiography or histological results. One animal was excluded from vasoreactivity testing due to denudation during vessel explant.

#### Bone marrow aspiration and seeding of the TEVG

Fifty-three lambs underwent bone marrow aspiration (5 mL/kg body weight) and implantation of an autologous cell-seeded TEVG as an intrathoracic IVC interposition graft. Animals were anesthetized using propofol (5 mg/kg) for induction and isoflurane (1–4%) or propofol (20–40 mg/kg/h) for maintenance. Lambs were placed in the lateral recumbent position, and the area overlying the iliac crest was shaved and prepped in standard sterile fashion. A 5-mm incision was made and an aspiration needle was inserted into the bone. Heparinized syringes (20 mL, 100 U/mL) were used to aspirate bone marrow. Following aspiration, the bone marrow was processed using Ficoll density gradient centrifugation or Pall filtration to isolate the bone marrow-derived mononuclear cells as previously described^35^. Briefly, for Ficoll centrifugation, bone marrow was filtered through 100 μm cell strainers to remove bone spicules and clots. A 1:1 dilution was achieved with phosphate buffered saline (PBS) and the bone marrow was layered onto Ficoll 1077 (Sigma-Aldrich). The plasma and mononuclear cell layers were isolated after centrifugation. The mononuclear cell layer underwent two washes with PBS to yield a cell pellet that was diluted in 20 mL of PBS. For Pall filtration, bone marrow was similarly filtered through a strainer to remove bone spicules and clots, and was then diluted 1:1 with PBS and passed through a Pall filter (Cook) to catch mononuclear cells and allow red blood cells and platelets to pass through. Mononuclear cells were then collected from the filter through backflushing with PBS. Mononuclear cells were vacuum-seeded onto the scaffold which was incubated in autologous plasma until the time of implantation.

#### Surgery

The scaffolds were implanted in the intrathoracic IVC as previously described^10,33,34^. Lambs were placed in a left lateral recumbent position. Depending on each animal’s anatomy, a right thoracotomy was made in the fifth or sixth intercostal space, and the thoracic IVC was dissected between the diaphragm and right atrium. A cavoatrial shunt was placed to maintain perfusion during cross-clamping of the IVC. The vessel was clamped and a 2 cm segment of a diameter-matched, seeded scaffold was implanted; with end-to-end anastomoses performed using a running nonabsorbable monofilament suture. No native vessel was removed. Titanium vascular clips or radiopaque circumferential markers were applied to the suture tails to mark the anastomoses for post-operative imaging. The chest wall, overlying muscle, and skin layers were reapproximated with absorbable sutures.

#### Interventional imaging

Post-operative catheterizations were performed at 1, 6, 26, 52, 104, and 156 weeks. Additional imaging was performed as needed based on each animal’s clinical condition. After sedation and intubation, lambs were placed in a left lateral decubitus position. The right internal jugular vein was cannulated, and a 9-French sheath (Terumo Medical Corporation, Somerset, NJ) was inserted followed by an intravenous bolus of heparin (150 U/kg). A 5 French JR 2.5 catheter (Cook Medical, Bloomington, IN) was passed into the right internal jugular vein through the SVC and into the right atrium. Using an angled Glidewire (Terumo Medical Corporation), the JR catheter was then passed through the TEVG into the intraabdominal IVC where a Rosen exchange guidewire (Cook Medical) was placed. A digital angiogram was then obtained by injecting ioversol 68% through the multitrack angiographic catheter positioned in the intraabdominal IVC. Diameters were measured at seven points: the intraabdominal IVC, low intrathoracic IVC (on the diaphragmatic side of the TEVG), proximal anastomosis (defined with respect to blood flow), mid-graft, distal anastomosis, high intrathoracic IVC (on the atrial side of the TEVG), and the area of most severe narrowing. The proximal and distal anastomoses were identified by the aforementioned surgically-placed radiopaque clips. An intravascular ultrasound catheter (Volcano Corporation, San Diego, CA) (IVUS) was advanced through the graft over the Rosen guidewire to obtain images at the same seven points measured during angiography. These images were analyzed using Volcano software to obtain a cross-sectional area as described previously^34^. Neotissue deposition within the TEVG wall was measured by using IVUS to quantify thickness. Representative angiography videos from a single animal over two years post-implantation shown in Supplemental Video 1.

#### Euthanasia

At the prescribed endpoint, animals were deeply sedated with ketamine (20 mg/kg) and diazepam (0.02–0.08 mg/kg) followed by induction of bilateral pneumothoraxes and exsanguination. A complete veterinary necropsy was performed at the time of TEVG explantation. Animals were also euthanized if they developed clinically significant stenosis (*n* = 2), defined here as graft narrowing with systemic symptoms. Animals that were not euthanized for clinically significant stenosis were euthanized at 6 weeks (*n* = 12), 6 months (*n* = 10), or 12 months (*n* = 12) post-implantation. The remaining animals were kept for long-term follow up, with *n* = 3 euthanized at 18 months for late-term mechanical testing.

### Histology

TEVGs and adjacent IVC tissue were explanted, fixed with 4% formalin for 1 week, then transferred to 70% ethanol for long-term storage. Upon removal from ethanol, explants were cut into smaller pieces to facilitate paraffin embedding and enable histological sections to be prepared within the proximal IVC, the TEVG near the proximal anastomosis, mid-graft, and near the distal anastomosis within the TEVG, as well as within the distal IVC. 4 µm transverse sections were mounted on slides and heat fixed. Standard techniques were adopted for hematoxylin and eosin, Picro-Sirius Red, and Masson’s Trichrome. Immunohistochemistry was used to detect the antigens listed in Supplemental Table 1. Samples underwent heat-induced antigen retrieval with Dako target retrieval solution in a pressure cooker using either citrate buffer (pH 6.0) or Tris-EDTA buffer (pH 9.0) followed by blocking for endogenous peroxidase (3.0% H_2_O_2_ in H_2_O) and non-specific binding (3% normal goat serum in Background Sniper, BioCare Medical). After primary antibody incubation overnight at 4C, sections were incubated sequentially in appropriate biotinylated secondary antibodies (1:1500, Vector) and streptavidin-horseradish peroxidase (Vector). DAB+ substrate chromogen (Vector) was used for color development. All samples were counterstained with Gill’s hematoxylin (Vector) prior to dehydration and cover-slipping.

Histomorphometric analysis of stenosis was performed in ImageJ (NIH, MD, USA). Boundaries between the neoadventitia and the outer surface of the scaffold, between the inner surface of the scaffold and the neointima, as well as the luminal surface were traced. Area and perimeter values for these boundary lines were used to calculate the luminal area remaining as compared to a scaffold at implantation, neointimal area, and the fold-increase in scaffold cross-sectional area. These values were then used to estimate factors that contribute to overall stenosis including intramural growth and inward remodeling.

Histological images were analyzed with ImageJ by users blinded to experimental time point. Positive areas were measured using pixel specific thresholding of hue, saturation, and lightness (HSL). For collagen fiber thickness ratios, measured using PSR under linear polarized light, thick fibers were classified by red/orange pixels and thin fibers by green/yellow.

### Accelerated scaffold degradation

Five mm long rings were cut from 16 mm diameter TEVGs, and dry weight was measured. Samples were submerged in 1x PBS heated to 70 °C for 0, 1, 3, 5, 7, 9, and 14 days, washed twice with ddH_2_O, frozen to −80 °C, lyophilized overnight, and remaining dry mass was measured. Zero-day control specimens were submerged in PBS for 5 min before washing and lyophilization to account for processing effects.

Mechanical testing was performed on a 100 Series TestResources MTI with a 10 N load cell at a tensile rate of 3 mm per second. Tensile load was equated to equivalent pressure through: *P* = mg/(2*Zr*), where *P* is the pressure, *m* is the hanging mass, *g* is the gravity, *Z* is the initial axial length of sample, and *r* is the initial internal radius of sample. Burst pressure was defined by the equated pressure which caused sample failure, or defined as 0 mmHg for samples which did not have enough structural integrity to load into the tester, or >1000 mmHg for samples which did not break under the maximum force of the load cell (equivalent to 1000 mmHg).

Specimens for microstructural analysis were placed onto SEM mounts with carbon tape, and a subset had the outer PCLA sponge surface removed using forceps under a dissecting microscope to reveal the inner PGA fiber layer. Samples were sputter-coated with gold under argon vacuum to 3 nm and imaged on a Hitachi S4800 SEM at 5 kV and 10 mA. SEM images were analyzed with FIJI image analysis software (NIH, MD, USA). Pore size was calculated by 7 SEM images at 100x of sample lumens. Fiber diameter was calculated by an average of at least 5 PGA fibers.

Samples of the rings were cut and dissolved at 1 mg/mL in 50 mM NaOH for 48 h at 80C to ensure complete degradation. Aliquots of dissolved solutions were diluted 1/10 in 50 mM NaOH and separately analyzed for lactate, a degradation product of PCLA, and glycolic acid, the degradation product of PGA. Lactate was measured using a commercially available Lactate Assay (Sigma-Aldrich, MO, USA). Glycolic acid was analyzed using a method adapted from Takahashi, 1972^36^. Mixed ratios of dissolved pure PCLA and pure PGA were used to generate standard curves.

### Ex vivo mechanical testing

The TEVGs were excised with the adjacent thoracic IVC. The perivascular tissue was gently removed, and the composite vessel-graft construct was mounted on custom plastic cannulas in Hank’s Balanced Salt Solution. The composite construct was secured to the cannula at the atrium and the diaphragm junctions using 3-0 sutures. Tubular biaxial testing was performed using a computer-controlled device^37^. Force and pressure were measured using standard transducers, diameter was tracked with an optical video-scope, and length was prescribed using a stepper motor. The vessel was initially equilibrated at ~5 mmHg and preconditioned with six cycles of pressurization from 0 to 30 mmHg, at in vivo stretch; the stretch at which axial force is approximately a constant with change in pressure. The biaxial protocol has a total of seven tests, three pressure-distension tests (1–30 mmHg) at constant axial stretches and four axial force-extension tests at constant pressures, details of which can be found elsewhere^11^. Circumferential stress-stretch behaviors from the pressure-distension test at in vivo stretch are reported for the 6-week and 78-week samples as well as the native IVC.

### Magnetic resonance imaging

Animals were evaluated at 1, 6, and 52 weeks post-implantation using a Siemens Plasma 3 T MRI (Siemens Medical Solutions, Erlangen, Germany). Animals were sedated with propofol and intubated for all MRI imaging studies. After acquisition of initial scout images, native IVC proximal and distal to the graft, proximal and distal anastomotic sites, and the mid-graft were analyzed using black blood fast spin echo MRI, contrast enhanced 3D MR angiography (MRA) MR 2D and 3D flow velocity mapping, and delayed enhancement imaging for fibrosis, and compared between 1, 6, and 52 weeks. Patterns of luminal distortion were assessed and characterized using flow velocity changes and tissue response as demonstrated on dynamic early contrast enhancement (DCE) and delayed enhancement (DE). Representative MRI videos at 1 and 52 weeks shown in Supplemental Video 2.

### Computational modeling: fluid–structure interactions

Subject-specific 3D geometries at 1, 6, and 52 weeks post-TEVG implantation were generated by importing MRI images into SimVascular^38^. Wall thickness was generated from intravascular ultrasound measurements and further edited using Meshmixer (Autodesk, Inc.). Blood was treated as an incompressible Newtonian fluid with constant hemodynamic properties (ρ = 1060 kg/m^3^, μ = 4.0 × 10^−3^ Pa s). We accounted for large vessel wall-deformations through the arbitrary Lagrangian–Eulerian formulation, where the fluid is governed by the Navier–Stokes equations and two-way coupling exists between the fluid forces and vessel wall traction forces. The TetGen mesh generator was used in SimVascular to create an unstructured high-resolution mesh, where fluid and solid domain nodes coincided at the luminal boundary to satisfy the dynamic boundary conditions necessary for the ALE coupling^39^. Mesh sensitivity analysis was conducted on one geometry each time to ensure the numerical solutions were insensitive to further mesh refinements. Wall tissue was modeled as a Neo–Hookean material, and material properties of the IVC and TEVG were tuned to match in vivo deformations over the cardiac cycle. To represent the downstream vasculature, we prescribed a three-element Windkessel boundary condition at the outlet, with parameter values tuned to match in vivo pressure measurements from cardiac catheterization. We obtained inlet flow waveforms from PC-MRI and imposed these as inlet flow boundary conditions for each animal with a parabolic spatial profile.

### Vasoreactivity testing

TEVG, adjacent thoracic IVC, and native thoracic IVC (*n* = 3 per group), just inferior to the graft, were dissected from the same lamb and placed in ice-cold Krebs buffer containing (mM): NaCl, 118; KCl, 4.7; KH_2_PO_4_, 1.18; MgSO_4_ ∙ 7H_2_O, 1.64; NaHCO_3_, 25.0; Glucose, 5.55; Na-Pyruvate, 2.0; CaCl_2_ ∙ 2H_2_O, 2.52; (pH = 7.4). The vessels were cut into rings approximately 5 mm in height and mounted in a tissue bath system (Radnoti LLC, Covina, CA) containing 15 mL Krebs (37 °C, 95:5% O_2_:CO_2_) at 0.5 g of resting tension. Force was acquired using isometric force transducers (Radnoti) connected to a PowerLab 16/30 (AD Instruments, Colorado Springs, Colorado). Data were recorded using LabChart 7 (AD Instruments). After a one-hour equilibration period, the viability of the vessels was tested using 60 mM KCl. Following washing and return to baseline tension, the vessels were pre-constricted with 1 nM endothelin-1 (ET-1, Sigma-Aldrich, St. Louis, MO)^40^. Following pre-constriction, endothelial-dependent and endothelium-independent relaxation was assessed by adding increasing concentrations (10^−9^ M to 10^−4^ M) of acetylcholine (ACh, Sigma) and sodium nitroprusside (SNP, Sigma), respectively. After testing vessel dilatory capacity, the vessels were then subjected to cumulative concentrations of ET-1 (10^−12^ M to 10^−8^ M) to assess their ability to contract. Relaxation responses were plotted as a percentage of the ET-1 induced contraction for each vessel, and contraction to ET-1 was plotted as a percentage of the maximal response to 60 mM KCl. Concentration response data were fit to a log function, and log EC50 values were calculated by nonlinear regression analysis using GraphPad Prism 7.0 software (GraphPad, La Jolla, CA). The pharmacologically-tested samples were imaged under SEM as well as *en face* immunofluorescent staining with CD31 and eNOS to evaluate the endothelial cells lining the lumen of the neovessel. One sample was excluded due to denudation of the endothelial layer during explant.

### Construction of 3D TEVG geometries

TEVG boundaries were defined on 3D angiographic CT data using radiopaque markers at distal and proximal anastomoses that remained visible for the duration of the imaging study. Reconstruction of the TEVG within these borders was performed using the open source software Simvascular (www.simvascular.org)^38^. 3D TEVG geometry was reconstructed through an image segmentation technique, and the final 3D TEVG geometry was discretized and exported to an opensource data analysis and visualization software to determine volume (ParaView, www.paraview.org).

### Statistics and reproducibility

Bench-top degradation testing studies were performed with four independent samples as replicates for each time point. Mechanical testing of TEVG polymers was performed with three independent samples as replicates for each time point. For experiments performed with animal specimens (in vivo imaging, growth data, IHC analysis) each individual animal was considered as a replicate. Statistical analyses and linear regressions were performed using GraphPad Prism 7.03 (GraphPad Software Inc.). For histological and bench-top analyses, groups were compared using ANOVAs with post-hoc Tukey for multiple comparisons, except in cases of unequal variances, where nonparametric Mann-Whitney tests were performed, as noted in results. Linear regressions were performed utilizing histological staining as the independent variable, with histomorphometric measures of intramural growth and inward remodeling on identical animals as the dependent variable. A *p* value of 0.05 was considered significant.

## Results

### Computational model of G&R

An analysis of neotissue kinetics using a computational G&R model enables long-term predictions of TEVG behaviors. We previously developed and validated a constrained mixture based G&R model that accurately described and predicted native vessel behavior by simulating the evolving mass density of native constituents (*nat*) at time *s* according to

3 $${\rho }^{{{{{{\rm{nat}}}}}}}(s)={\int }_{0}^{s}{m}_{h}^{{{{{{\rm{mech}}}}}}}(1+{\varUpsilon }^{{{{{{\rm{mech}}}}}}}(\tau )){q}^{{{{{{\rm{mech}}}}}}}(s,\tau ){{{{{\rm{d}}}}}}\tau ={\int }_{0}^{s}{M}^{{{{{{\rm{nat}}}}}}}(s,\tau ){{{{{\rm{d}}}}}}\tau ,$$

where ${m}_{h}^{{{{{{\rm{mech}}}}}}}$ is the rate of mass density production of the native constituent in the homeostatic state, ${\varUpsilon }^{{{{{{\rm{mech}}}}}}}(\tau )$ is a stimulus function at time *τ* that modulates production according to deviations from the homeostatic values of intramural stress and wall shear stress, and ${q}^{{{{{{\rm{mech}}}}}}}(s,\tau )\in [0,1]$ is a survival function that tracks the degradation of a cohort produced at past time *τ* to current time *s*^11,41^. We subsequently modified this native vessel model to simulate G&R in TEVGs

4 $${\rho }^{{{{{{\rm{neo}}}}}}}(s)={\rho }^{{{{{{\rm{infl}}}}}}}(s)+{\rho }^{{{{{{\rm{mech}}}}}}}(s),$$

where *ρ*^neo^(*s*) is the total evolving mass density of all neotissue constituents, resulting from *ρ*^infl^(*s*) and *ρ*^mech^(*s*), that is, constituents produced in response to inflammatory stimuli and mechanical stimuli, respectively^11^. The model thus built upon our original description of native vessel G&R but with the addition of inflammation-mediated contributions induced by the polymeric scaffold, which we had shown experimentally to be critical for neotissue formation^11,42,43^. The current computational G&R model thus highlights the roles of both mechanical and inflammatory stimuli in driving the natural history of the TEVG. The mass density of mechano-mediated constituents was written as

5 $${\rho }^{{{{{{\rm{mech}}}}}}}(s)={\int }_{0}^{s}{M}^{{{{{{\rm{nat}}}}}}}(\tau )(1-\exp (-\tau )){{{{{\rm{d}}}}}}\tau ,$$

where ${M}^{{{{{{\rm{nat}}}}}}}(\tau )$ describes the native constituent kinetics at each time *s* from Eq. (3), and an exponential-decay term modifies the time of its influence to account for delayed cellular infiltration. Importantly, the model considers that the scaffold alters G&R via stress-shielding, where decreased intramural stresses from the presence of a stiff polymer can decrease mass density production as in a native vessel subjected to reduced loading^44^. Additionally, the luminal wall shear stress changes as the diameter of the TEVG evolves. With the stenosis observed in early remodeling, wall shear stress increased and intramural stress decreased, both acting as mechanobiological stimuli to decrease mass density production via the mechano-mediated stimulus function ${\varUpsilon }^{{{{{{\rm{mech}}}}}}}(\tau ) \, < \, 0$ in the early remodeling phase. Inflammatory effects from the foreign body response generated a new class of constituents whose mass density were modeled as

6 $${\rho }^{{{{{{\rm{infl}}}}}}}(s)={\int }_{0}^{s}{m}_{h}^{{{{{{\rm{infl}}}}}}}({\varUpsilon }^{{{{{{\rm{infl}}}}}}}(\tau ))(1-{{{{{\rm{exp}}}}}}(-\tau )){q}^{{{{{{\rm{infl}}}}}}}(s,\tau ){{{{{\rm{d}}}}}}\tau ,$$

where ${m}_{h}^{{{{{{\rm{infl}}}}}}}$ is a basal rate of inflammatory production, ${\varUpsilon }^{{{{{{\rm{infl}}}}}}}(\tau )$ is a gamma function accounting for the transient production of immuno-mediated neotissue until polymer degradation is complete, and ${q}^{{{{{{\rm{infl}}}}}}}(s,\tau )\in [0,1]$ is the survival function for the cohort produced at past time *τ* that persists at current time *s*. With the assumption that complete polymer degradation leads to eventual resolution of the immune response, we can write ${\rho }^{{{{{{\rm{infl}}}}}}}(s\to \infty )=0$. Then, since ${\rho }^{{{{{{\rm{mech}}}}}}}(s\to \infty )={\rho }^{{{{{{\rm{nat}}}}}}}(s)$ with the exponential decay term approaching zero,

7 $${\rho }^{{{{{{\rm{neo}}}}}}}(s\to \infty )={\rho }^{{{{{{\rm{infl}}}}}}}(s\to \infty )+{\rho }^{{{{{{\rm{mech}}}}}}}(s\to \infty )={\rho }^{{{{{{\rm{nat}}}}}}}(s)$$

Thus, the behavior of the TEVG can be separated into two periods: the first, hereafter referred to as the period of neotissue formation, during which G&R are mainly influenced by the immune response to the scaffold material and, the second, referred to as the period of neovessel remodeling, which occurs after scaffold degradation when the mechano-mediated stimulus ${\varUpsilon }^{{{{{{\rm{mech}}}}}}}(\tau )\, > \,0$ due to increasing intramural stress and decreasing wall shear stress with decreasing thickness and increasing diameter. Consequently, a key result of the model is the prediction that neovessel G&R should eventually mimic the purely mechano-mediated G&R found in native vessels. Further description of the G&R model can be found in the “Methods”.

### Natural history of neovessel formation

**Fig. 1:**
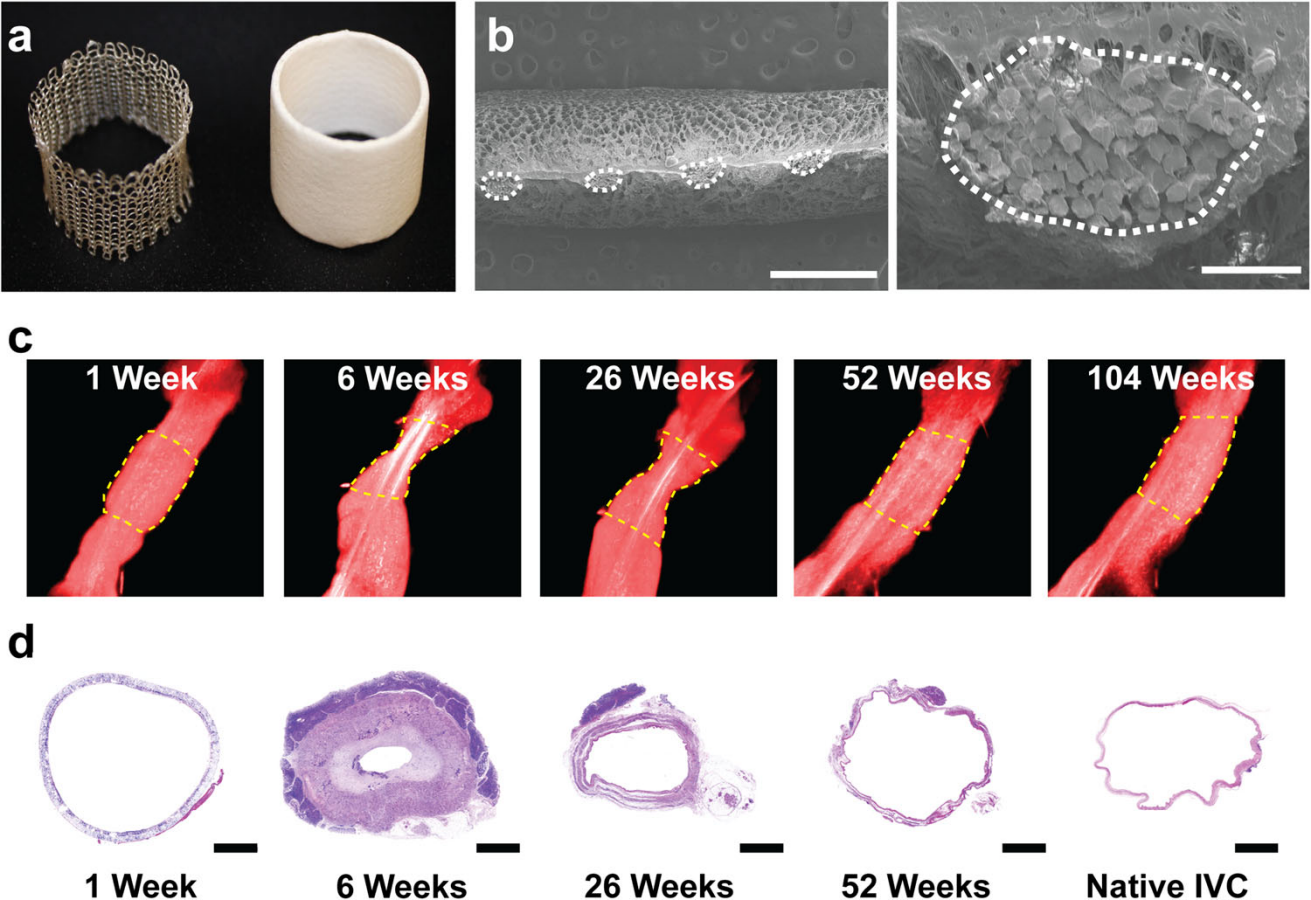
Natural history of tissue engineered vascular graft development. **A** TEVG scaffold was fabricated from PGA fibers that were knitted into a tube (left) and coated on the inner and outer surface with a PCLA (right). **B** Magnified SEM images of the scaffold demonstrated sponge layers of PCLA surrounding the PGA fibers outlined with a white dotted line. Scale bars 1 mm left, 100 µm right. **C** Representative 3D reconstructions of a TEVG (outlined in yellow) over its 2-year implantation as an IVC interposition graft in a sheep model. **D** Representative histologic H&E images demonstrated characteristic changes in TEVG over the 1-year time course. Scale bar 4 mm. TEVG: tissue engineered vascular graft, PGA: polyglycolic acid, PCLA: polycaprolactone-lactide, SEM: scanning electron microscope, IVC: inferior vena cava.

Scaffolds were fabricated from polyglycolic acid (PGA) fibers that were knitted into a tube and coated with a 50:50 copolymer of polycaprolactone and polylactide (PCLA), which together formed a porous sponge (Fig. 1A, B). The scaffold was designed to degrade by hydrolysis. Our previous studies demonstrated that upon implantation, the scaffold initially functioned as a synthetic vascular conduit; soon thereafter, neotissue formed as the scaffold degraded. Neotissue formation, as characterized by angiography and histology, was highly dynamic over the first 26 weeks after implantation, with marked graft narrowing during the first six weeks followed by spontaneous reversal by 26 weeks^11^. Beyond the initial 26-week period, morphological changes were more gradual (Fig. 1C), similar to native vessel G&R under nearly constant hemodynamics. Once the scaffold degraded completely, by 52 weeks, the resulting neovessel grossly resembled a native blood vessel (Fig. 1D).

### Mechanisms underlying G&R

**Fig. 2:**
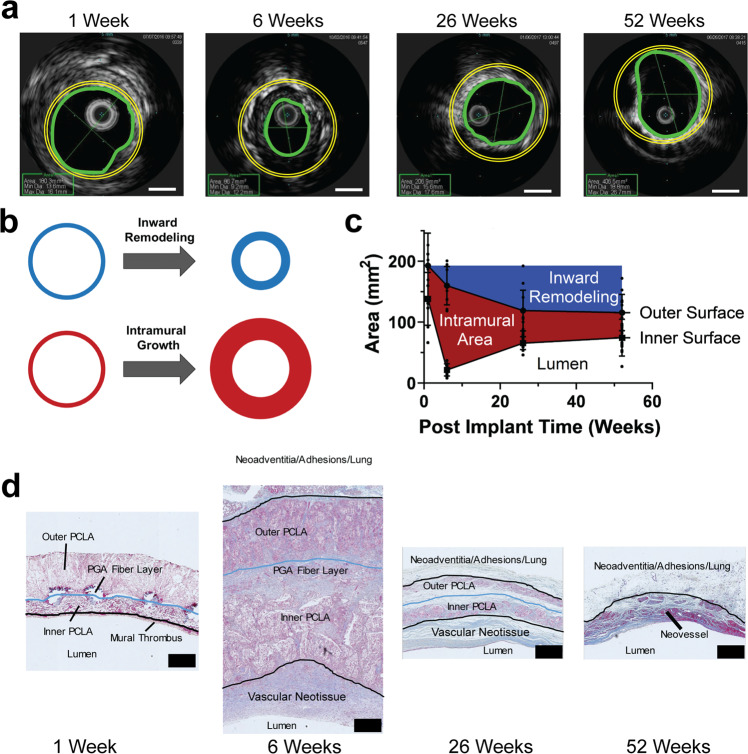
Morphometric changes during neotissue formation and development. **A** Representative intravascular ultrasound (IVUS) imaging of TEVG, with lumen outlined in green and original TEVG size overlayed for reference in yellow. Scale bar 5 mm. **B** Remodeling in TEVGs occurred through two main processes, inward remodeling (blue) with a decrease of outer diameter, and intramural growth (red) with a thickening of the vessel wall. **C** Quantification of changes in inner and outer diameter of TEVGs in the sheep model measured by IVUS. **D** High magnification representative trichrome staining demonstrated intramural growth via inflammatory tissue formation and vascular neotissue formation, followed by subsequent mural thinning as the scaffold degraded and the inflammatory neotissue subsided resulting in the creation of a neovessel. Scale bar 500 µm. Data shown as mean+/-SD. IVUS: intravascular ultrasound, TEVG: tissue-engineered vascular graft.

Serial intravascular ultrasound (IVUS) imaging over the 52-week study period (1, 6, 26, and 52 weeks post-implantation) provided additional information on the morphology of the evolving TEVG (Fig. 2A). Changes in the lumen of a blood vessel can arise from (i) thickening or thinning of the wall (intramural growth defined here as an increase in thickness of the graft wall), (ii) inward or outward remodeling (inward remodeling defined here as an inward change in the outer wall of the graft), and (iii) combinations of the two (Fig. 2B). Serial IVUS data comparing wall thickness to luminal diameter demonstrated that the TEVG lumen narrowed between 1 and 6 weeks primarily due to wall thickening through intramural growth. IVUS imaging between 6 and 26 weeks revealed wall thinning in addition to inward remodeling, namely, a decrease in outer diameter without a decrease in inner diameter. Between 26 and 52 weeks, the wall continued to thin and the lumen expanded (Fig. 2C).

Histological evaluation of the explanted TEVGs demonstrated that between 1 and 6 weeks after implantation, the luminal narrowing causing TEVG stenosis was primarily due to thickening of the scaffold wall. H&E staining demonstrated that this thickening arose from the infiltration and proliferation of cells, resulting in TEVG wall area at 6 weeks being 371 ± 66% of the implanted scaffold wall area by IVUS. There was also evidence of appositional growth of vascular neotissue along the luminal surface of the scaffold, though it accounted for only 16 ± 5% of the total wall area at 6 weeks. The TEVG stenosis spontaneously reversed between 6 and 26 weeks as the wall thinned (IVUS wall thickness 6.5 ± 1.2 mm 6 week vs 2.7 ± 0.5 mm 26 week). This thinning arose from degradation of the scaffold and loss of associated neotissue, yet the lumen did not fully return to its pre-implant area due to persistent inward remodeling. The wall thinned further between 26 and 52 weeks with the lumen enlarging as the scaffold and its associated neotissue waned (Fig. 2D**)**.

Interestingly, the correlation between TEVG lumen area and wall thickness as measured by IVUS changed throughout the time course (Supplemental Fig. 1). At one week, there was no correlation between the two, as the graft parameters were primarily defined by the scaffold. At six weeks, the point of highest measured graft wall thickness, there was a negative relationship between IVUS lumen area and wall thickness, with thicker walls leading to more stenosis. After 26 weeks post-implantation, there again was no relationship; however, after 52 weeks, the relationship was positive, with larger lumens having thicker walls.

### Role of the scaffold on G&R (in vitro degradation study)

**Fig. 3:**
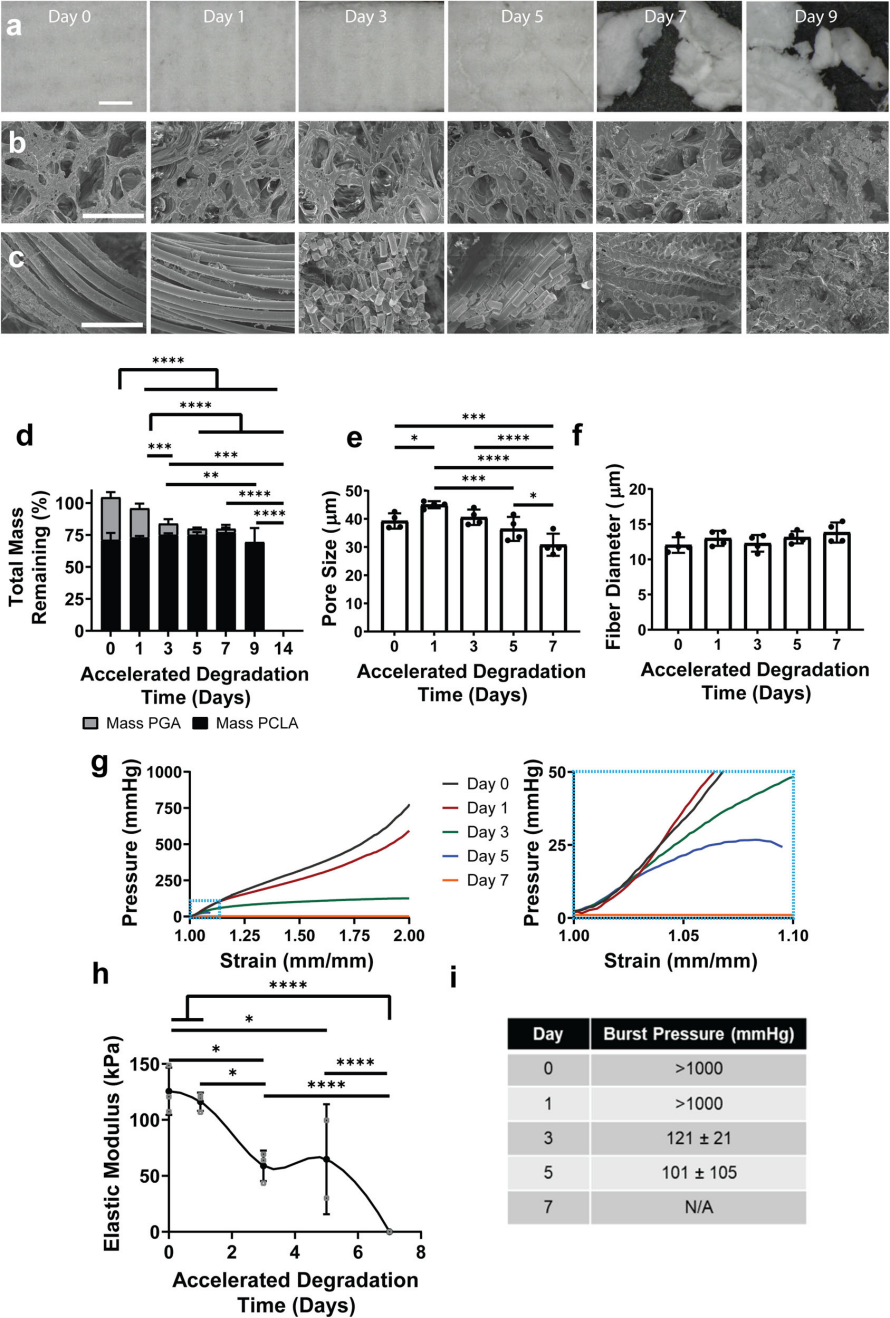
In vitro accelerated degradation of TEVG scaffolds. Accelerated degradation studies demonstrated a breakdown of the macrostructure (**A**) as well as PCLA (**B**) and PGA (**C**) microstructures. **D** Mass and polymer degradation of TEVGs, with PCLA pore (**E**) and fiber (**F**) sizes quantified. **G** Strain vs pressure curves of mechanical testing of TEVGs subjected to accelerated degradation studies (*N* = 3/time point), with blue-outlined low-pressure region magnified on right. Changes in elastic modulus (**H**) and burst pressure (**I**) quantified from mechanical testing. Scale bars (**A**) 1 mm, (**B**, **C**) 100 µm. Data shown as mean ± SD. Statistical significance determined using ANOVA with Tukey post-hoc test. *<0.05, **<0.01, ***<0.001, ****<0.0001. PCLA: polycaprolactone-lactide, PGA: polyglycolic acid, TEVG: tissue engineered vascular grafts.

Based on the computational G&R model prediction of the dual roles played by the scaffold, we characterized both its evolving material properties and diminishing mass, which provided stress shielding and inflammatory stimuli, respectively. The accelerated scaffold degradation study utilized the temperature-dependent reaction kinetics of hydrolytic degradation of the PGA/PCLA scaffolds by bathing samples for 0, 1, 3, 5, 7, 9, and 14 days in PBS heated to 70 °C in vitro (Fig. 3A–C). 70 °C was chosen as preliminary studies revealed that one day of accelerated degradation corresponded to approximately one month of real-time degradation at 37 °C (Supplemental Fig. 2). Quantitative analyses revealed complete degradation over 14 days in vitro, with differential rates of degradation for PGA (by day 5 in vitro, estimated month 5 in vivo) and PCLA (by day 14 in vitro, estimated month 14 in vivo) (Fig. 3D). The TEVG was constructed with 25% of the initial mass being the knit central PGA layer, with the remaining 75% of the initial mass representing the PCLA sponge layers. Morphometric characterization via SEM demonstrated that pore size initially increased and then gradually decreased (Fig. 3E) while fiber diameter persisted during the early period (0–5 days) (Fig. 3F) but changed dramatically as bulk erosion continued and the PGA knit was no longer apparent by SEM imaging or chemical analysis (7 days and beyond). Following loss of mechanical integrity of the PCLA sponge (at 7 days in vitro, estimated 7 months in vivo); the scaffold existed solely as small fragments that continued to degrade until no visible fragments remained at 14 days.

Results demonstrated further that the scaffold became progressively more compliant as it degraded (Fig. 3G). There was a sudden increase of compliance (i.e., decreasing slope of the pressure–strain curve) at 3 days in vitro (estimated 3 months in vivo), corresponding to loss of mechanical integrity of the PGA fibers (125 ± 21 kPa day 0, 116 ± 8 kPa day 1, 59 ± 14 kPa day 3; significantly different between days 1 and 3 based on a Tukey post-hoc test with *p* < 0.05) (Fig. 3H). By day 7, the scaffold lost mechanical integrity and could not withstand pressure (i.e. compliance approached infinity and stiffness decreased to zero) (65 ± 49 kPa day 5, 0 ± 0 kPa day 7). Similar findings emerged for equivalent burst pressure, which dropped precipitously at 3 days with the loss of PGA fiber integrity (>1000 kPa day 0 and day 1, 121 ± 21 kPa day 3, 101 ± 105 kPa day 5) and at 7 days the burst pressure was not measurably due to the scaffold being too degraded for testing with the loss of PCLA sponge integrity (Fig. 3I). Of note, from a physiologic perspective the scaffold remained relatively stiff compared to the native IVC over the physiologically relevant pressure range (1–0 mmHg) until after 5 days of accelerated degradation. Thus, these results suggested the change in inflammatory stimulation (presence of scaffold) and the stress-shielding properties (structural integrity of scaffold) are uncoupled, since the loss of structural integrity occurred well before the scaffold mass disappeared.

### Role of the scaffold on G&R (in vivo time course study)

**Fig. 4:**
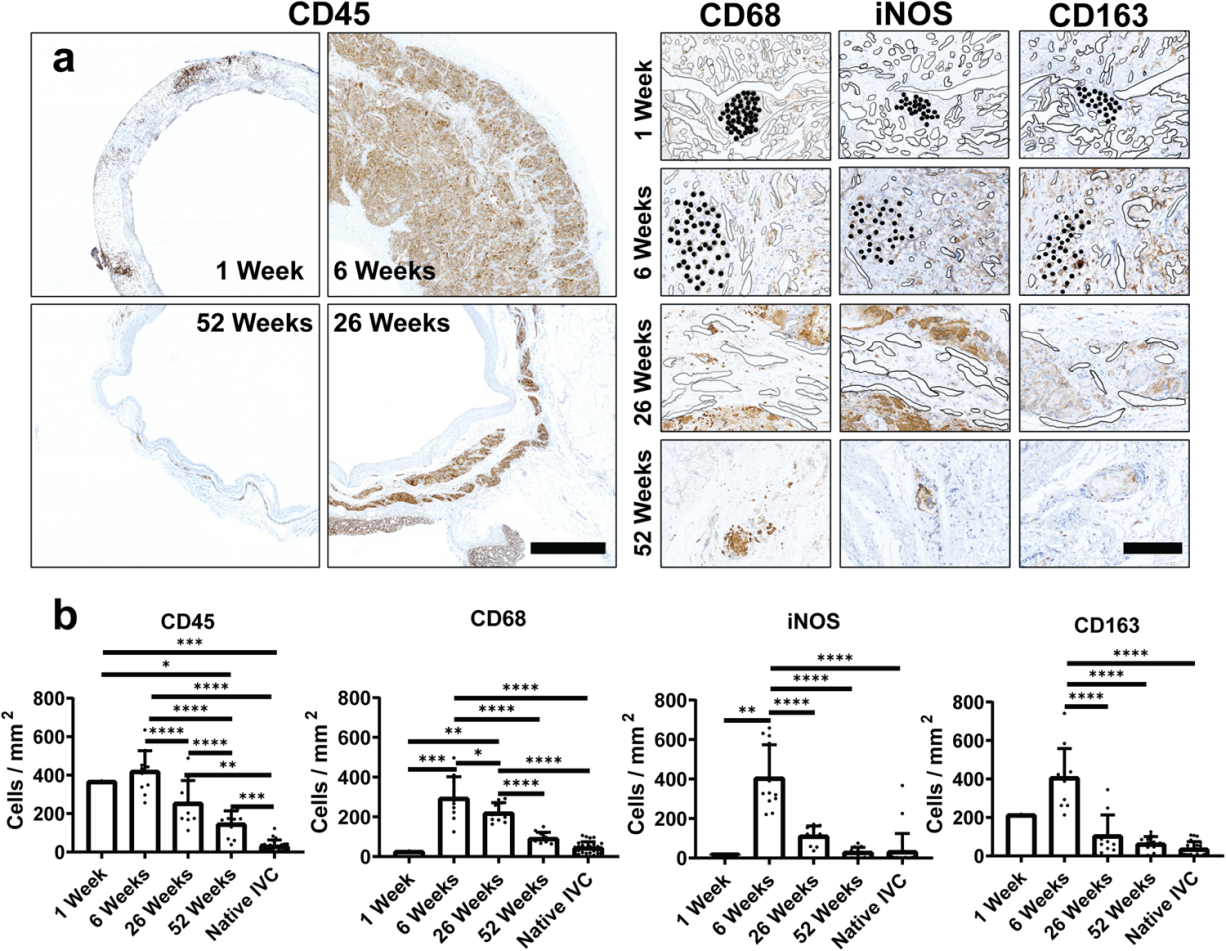
Inflammatory constituents throughout neovessel formation. **A** Representative histology of inflammatory cells, including CD45, CD68, iNOS, and CD163 over 1-year time course after implantation. PGA labeled in solid black, with PCLA outlined in black Scale bars left 2000 µm, right 200 µm. **B** Quantifications of histological inflammatory markers. Data shown as mean ± SD (*N* = 1, 12, 10, 12, 25 for 1 week, 6 week, 26 week, 52 week, and native respectively). Statistical significance determined using ANOVA with Tukey post-hoc test. *<0.05, **<0.01, ***<0.001, ****<0.0001. PGA: polyglycolic acid, PCLA: polycaprolactone-lactide.

Next we evaluated scaffold-induced inflammation in vivo. TEVGs implanted as sheep IVC interposition grafts were harvested at 1, 6, 26, and 52 weeks after implantation and characterized using immunohistochemical (IHC) stains (Fig. 4A). Markers of the inflammatory response, including CD45 for leukocytes and CD68 for monocytes and macrophages, were present within the neotissue, particularly at earlier time points. Histological evaluation demonstrated degradation of the scaffold in vivo mirrored its degradation in vitro, including differential rates of degradation, with PGA fibers (solid black) degrading prior to the PCLA sponge (black outline) between 6 and 26 weeks (Fig. 4B). However, there was scattered residual scaffold material and patches of inflammatory cells remaining at 52 weeks, emphasizing differences between polymer degradation in vivo and in vitro. Also, the thickness of the polymeric scaffold in vivo increased from the manufactured value of 0.70 mm at implant to 1.3 ± 0.3 mm at 1 week and 3.0 ± 0.6 mm at 6 weeks (1- vs 6-week Tukey adjusted *p* < 0.001) then decreased to 1.9 ± 0.4 mm by 26 weeks (6 vs 26 week Tukey adjusted *p* < 0.001). As this thickening did not occur in PBS in vitro, it was likely not due to polymer swelling. Rather, cell infiltration and extracellular matrix (ECM) accumulation (inflammatory neotissue formation) accounted for most scaffold thickening observed following implantation.

IHC staining delineated the inflammatory cells throughout TEVG development. CD45+ leukocyte populations and CD68+ monocytes and macrophages rapidly populate the scaffold, residing within the pores. The inflammatory stimulus, measured by CD45+ cell density, continued to rise until reaching the highest measured levels around 6 weeks (437 ± 91 cells/mm^2^), after which this density decreased (257 ± 110 cells/mm^2^ at 26 weeks, Tukey adjusted *p*-value vs 6 weeks *p* < 0.001), becoming closer to, but still higher than, the native background inflammatory cell density by 52 weeks (148 ± 63 cells/mm^2^ TEVG vs 34 ± 28 cells/mm^2^ IVC; 26- vs 52-week TEVGs, Tukey adjusted *p* < 0.01; 52-week TEVG vs IVCs, Tukey post-hoc *p* < 0.001). Pro-inflammatory (iNOS+) and anti-inflammatory (CD163+) macrophages and monocytes appeared to rise and fall in tandem throughout TEVG evolution. At 52 weeks, there were slightly higher levels of lingering anti-inflammatory compared to pro-inflammatory macrophages (31 ± 21 iNOS+ cells/mm^2^ vs 65 ± 34 CD163+ cells/mm^2^, t-test *p* = 0.008), likely related to long-term retention of low levels of foreign body giant cells within the neotissue.

**Fig. 5:**
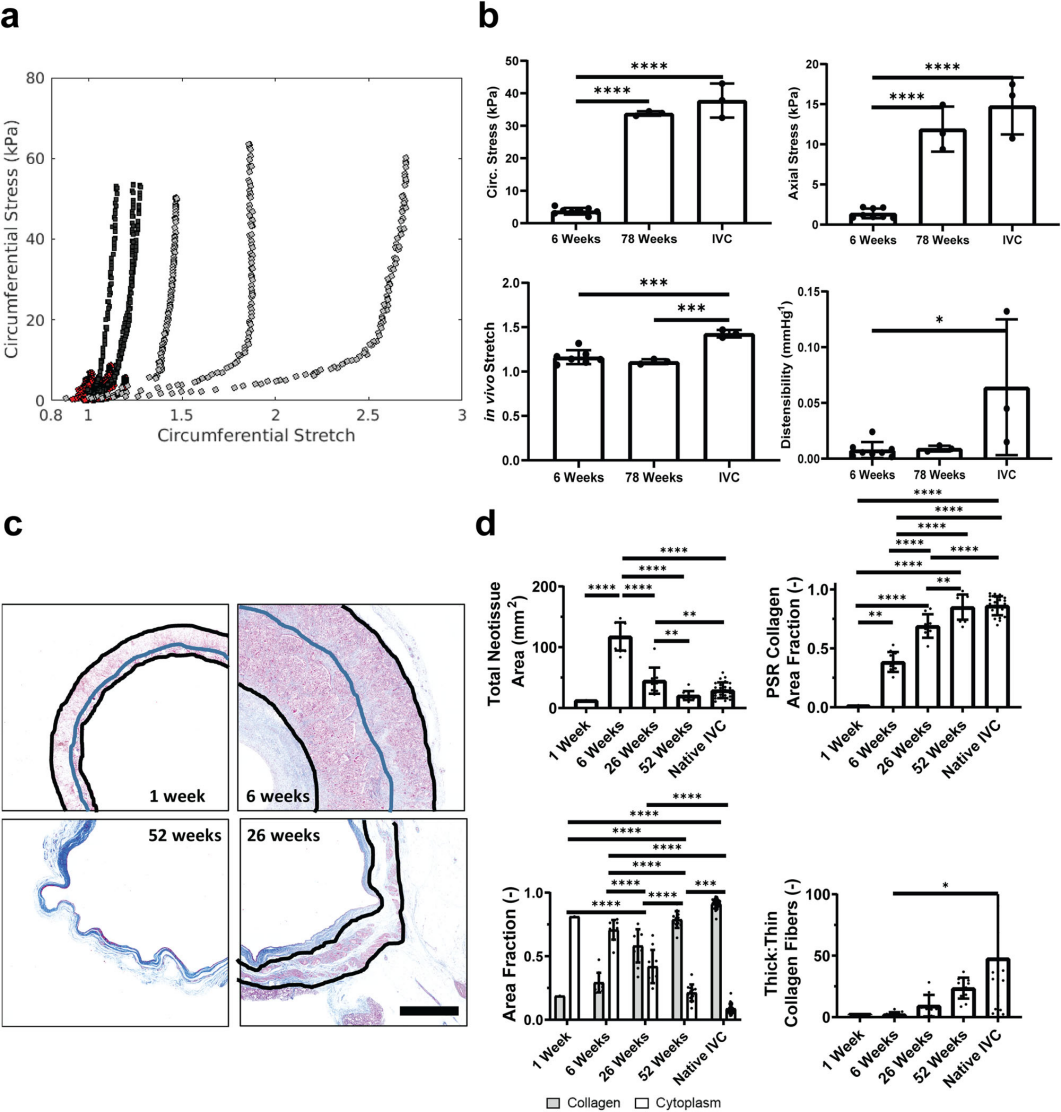
Mechanical constituents throughout neovessel formation. **A** Representative ex vivo biaxial mechanical testing of TEVGs at 6 weeks (red) (*N* = 8) and 78 weeks (black) (*N* = 3) as well as native IVC (white) (*N* = 3). Comparison of mechanical measurements from biaxial mechanical testing shown in (**B**). **C** Representative trichrome staining of TEVGs demonstrated changes in thickness as well as ECM and cellular composition of neotissue. PCLA layer outlined in black, with PGA layer noted in blue. D Quantifications of trichrome and Picro-Sirius Red staining. Scale bar 2 mm. Data shown as mean ± SD (histology *N* = 1, 12, 10, 12, 25 for 1 week, 6 weeks, 26 weeks, 52 weeks, and native respectively). Statistical significance determined using ANOVA with Tukey post-hoc test. *<0.05, **<0.01, ***<0.001, ****<0.0001. TEVG: tissue engineered vascular grafts, ECM: extracellular matrix, PCLA: polycaprolactone-lactide, PGA: polyglycolic acid.

We also evaluated the effect of the in vivo stress-shielding exhibited by the polymeric scaffold. Ex vivo biaxial mechanical testing of neovessels (Fig. 5A) demonstrated that at 6 weeks and 78 weeks the TEVG had a lower in vivo stretch than the native IVC (Fig. 5A) (1.17 ± 0.08 at 6 weeks, 1.11 ± 0.03 at 78 weeks, and 1.43 ± 0.04 for the IVC, Tukey adjusted *p*-value 0.515 for 6 vs 78 weeks, *p* = 0.00024 for 6 weeks vs IVC, and *p* = 0.00024 for 78 weeks vs IVC). The axial and circumferential wall stress were calculated at in vivo stretch and a representative pressure (10 mmHg for axial and 20 mmHg for circumferential). The axial wall stress (1.39 ± 0.60 kPa at 6 weeks, 11.89 ± 2.81 kPa at 78 weeks, and 14.77 ± 3.55 kPa for IVC, Tukey adjusted *p*-value < 0.0001 for 6- vs 78 weeks, *p* < 0.0001 for 6 weeks vs IVC, *p* = 0.224 for 78 weeks vs IVC) and the circumferential wall stress (3.71 ± 1.01 kPa at 6 weeks, 33.79 ± 0.69 kPa at 78 weeks, and 37.77 ± 5.26 kPa for the IVC, Tukey adjusted *p*-value < 0.0001 for 6- vs 78 weeks, *p* < 0.0001 for 6 weeks vs IVC, and *p* = 0.151 for 78 weeks vs IVC) was significantly lower for the 6 week group. Distensibility (0.0077 ± 0.0072 mm Hg^−1^ at 6 weeks, 0.0090 ± 0.0025 mm Hg^−1^ at 78 weeks, and 0.0640 ± 0.0609 mm Hg^−1^ for IVC, with Tukey adjusted *p*-value = 0.997 for 6 vs 78 weeks, *p* = 0.024 for 6 weeks vs IVC, *p* = 0.066 for 78 weeks vs IVC) of the TEVG was significantly lower than the native IVC at both 6 weeks and 78 weeks (Fig. 5B, Table 1). Axial and circumferential stress approached native IVC values at 78 weeks post-implantation, though the in vivo axial stretch and distensibility remained lower than the native IVC, suggestive of an altered matrix composition and deposition history.

**Table 1:** Mechanical testing.

	TEVG—6 weeks	TEVG—78 weeks	IVC
In vivo stretch	1.17 ± 0.08	1.11 ± 0.03	1.43 ± 0.04
Circumferential stress (kPa)	3.71 ± 1.01	33.79 ± 0.69	37.77 ± 5.26
Axial stress (kPa)	1.39 ± 0.60	11.89 ± 2.81	14.77 ± 3.55
Distensibility (mmHg^−1^)	0.0077 ± 0.0072	0.0090 ± 0.0025	0.0640 ± 0.0609

Comparison of mechanical metrics between tissue engineered vascular grafts (TEVG) at 6 weeks (*n* = 8) and 78 week (*n* = 3) with native inferior vena cava (IVC, *n* = 3). Data represents mean ± standard deviation.

The biomechanical properties of the TEVG arise from a combination of the material behavior of the scaffold and that of the neotissue constituents. As the polymer is initially very stiff relative to the neotissue, it bears most of the pressure-induced load. Hence, the material behavior of the TEVG derives almost exclusively from the scaffold shortly after implantation. Furthermore, the increased thickness of the TEVG wall relative to the native vessel thickness in combination with the presence of the stiff polymeric constituents reduce the intramural stresses experienced by the cells (Fig. 5B). As such, the cells are stress-shielded during the early remodeling process, but the degradation of the polymer and transfer of the load to the deposited ECM allows long-term mechanical loads to contribute to ECM remodeling towards a native-like structure. With marked stress-shielding through 6 weeks, the ECM is initially disorganized, but becomes the predominant load bearing constituent after polymer degradation. Thus, there is significant remodeling and maturation over time to form a highly ordered structure (Fig. 5C, D), likely due to mechano-mediated G&R predicted by modeling.

The total area of neotissue, measured from the trichrome stain, increased up to the 6 week time point (12.7 mm^2^ at 1 week vs 138.0 ± 37.7 mm^2^ at 6 weeks), when the inflammation was at its highest recorded levels, then decreased as the inflammation waned and the stenosis self-resolved (65.4 ± 31.9 mm^2^ at 26 weeks vs 30.3 ± 11.2 mm^2^ at 52 weeks, Tukey adjusted *p* = 0.003). Cell content was high (81%) 1 week after implantation whereas collagen content was low (19%), as measured by trichrome staining. This ratio reversed over time, approaching at 52 weeks the low cellularity, high collagen content (78.8 ± 6.6% TEVG vs 91.3 ± 4.8% IVC trichrome collagen area, Tukey adjusted *p* < 0.001) of the native IVC. Picro-Sirius Red staining showed nearly the same collagen content in the TEVG at 52 weeks as the native IVC (85.0 ± 10.6% TEVG vs 86.5 ± 8.0% IVC, Tukey adjusted *p* = 0.649). The ratio of thick to thinner collagen fibers also changed as the TEVG evolved, with thicker (mainly type I) collagen fibers becoming an increasingly larger percentage of total collagen at later times. This ratio remained lower than that seen in the native IVC at 52 weeks (thick:thin of 23.5 ± 8.5 for the TEVG vs 43.3 ± 52.2 for the IVC, Tukey adjusted *p* = 0.224), although there was variation in the ratios for the IVC samples.

### Hemodynamic changes

**Fig. 6:**
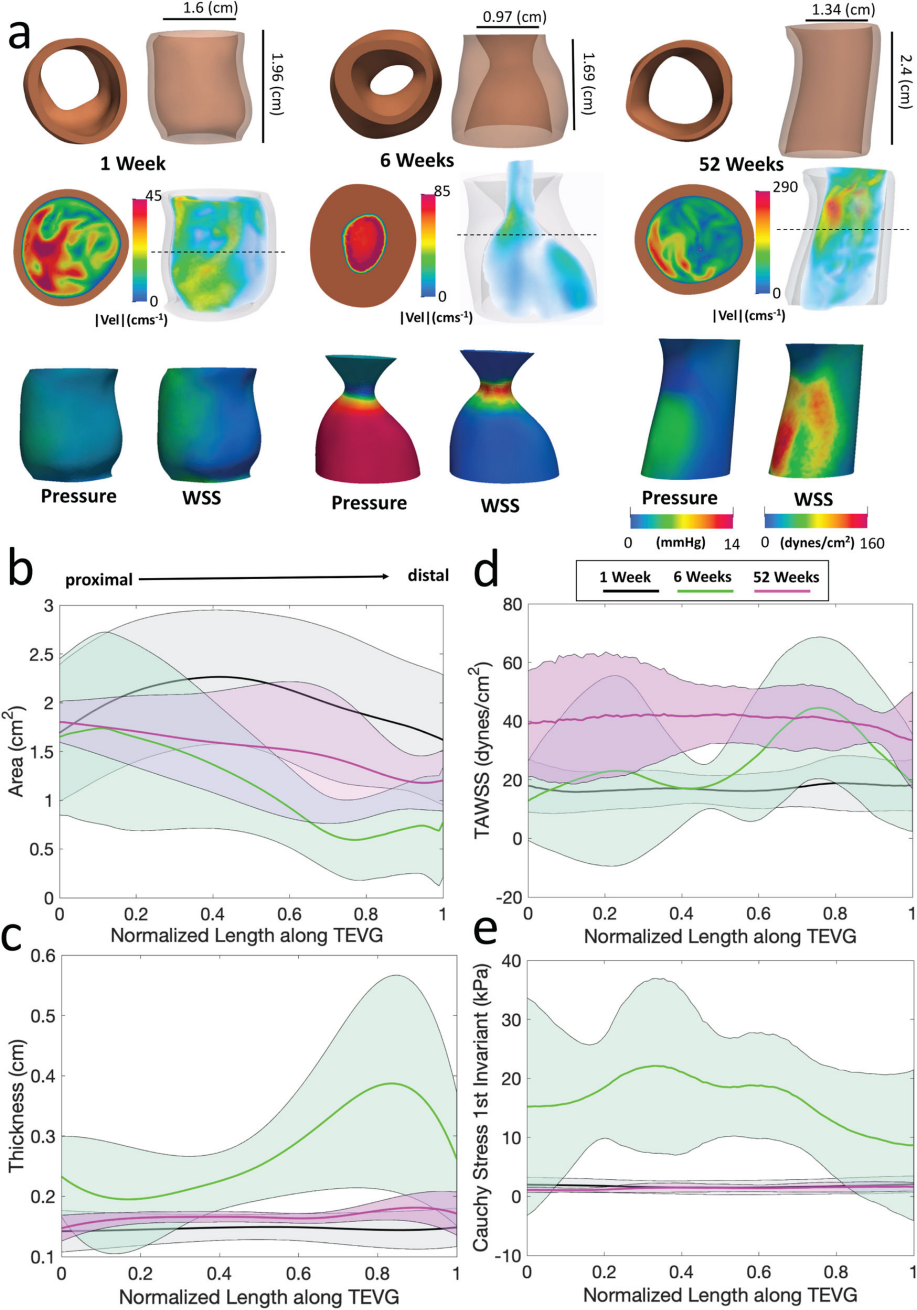
Hemodynamic changes throughout neovessel formation. **A** 3D anatomical models of TEVGs at 1-week, 6 weeks and 52 weeks post-TEVG implantation with representative velocity magnitude (peak flow), pressure, and wall shear stress maps (averaged over the cardiac cycle), as measured by FSI simulations. A corresponding cross-section shows flow through a slice of the TEVG volume (dotted line) and its corresponding luminal thickness. Note the increase in velocity magnitude across time points, the decrease in diameter and length from 1-week to 6 weeks and the flow patterns that were a result of geometric changes. Average ± standard deviation of lumen cross-sectional area (**B**), vessel wall thickness (**C**), time averaged wall shear stress (**D**), and Cauchy Stress (averaged over the cardiac cycle) (**E**) shown along the normalized length of the graft for each time point. Proximal to distal arrow indicates direction of flow. TEVG: tissue-engineered vascular grafts, FSI: fluid–structure interaction.

To assess effects of the evolving TEVG on hemodynamics and vice versa, we performed subject-specific computational simulations and calculated 3D maps of major hemodynamic indices. 3D anatomical models of TEVGs at 1, 6, and 52 weeks post-implantation (Fig. 6A) detailed structural changes. Subject-specific computational fluid–structure interaction (FSI) simulations in the respective geometries allowed calculation of velocity, pressure, and wall shear stress (WSS) and enabled in-depth spatio-temporal comparisons of morphological and hemodynamic parameters. Simulations highlighted characteristic changes seen in the natural history of TEVG development in vivo, from the cinching of the graft and IVC at the anastomoses to development of stenosis, evidenced by marked narrowing of the lumen cross-sectional area (Fig. 6B) and thickening of the wall (Fig. 6C), which reversed by 52 weeks. Effects of these morphological changes on hemodynamics are seen in (Fig. 6D, E**)**, where the conical-like 6 week geometry (from proximal anastomosis to stenosis) caused a spike in WSS at its focal point (Fig. 6D). Time-averaged wall shear stress (TAWSS) along the TEVG generally increased from 1 to 6 weeks, particularly in the stenotic region, and again from 6 to 52 weeks, as the TEVG elongated and narrowed. Effects of pressure on the vessel wall were quantified using the Cauchy stress (Fig. 6E). Given the Laplace estimation of circumferential Cauchy stress for a thin-walled cylinder (*Pr*/*h*), that is, luminal pressure multiplied by radius and divided by wall thickness, the large increase in Cauchy stress from 1 to 6 weeks arose in part due to the dramatic increase in pressure at 6 weeks. Cauchy stress subsequently decreased from its 6-week level back to the 1-week level by 52 weeks of implantation. The 1- and 52-week values were indicative of how stiff the TEVG was compared to the IVC. Further quantifications of MRI measurements given in Supplemental Fig. 3.

### Computational G&R analysis of inflammation-driven, mechano-mediated neotissue

**Fig. 7:**
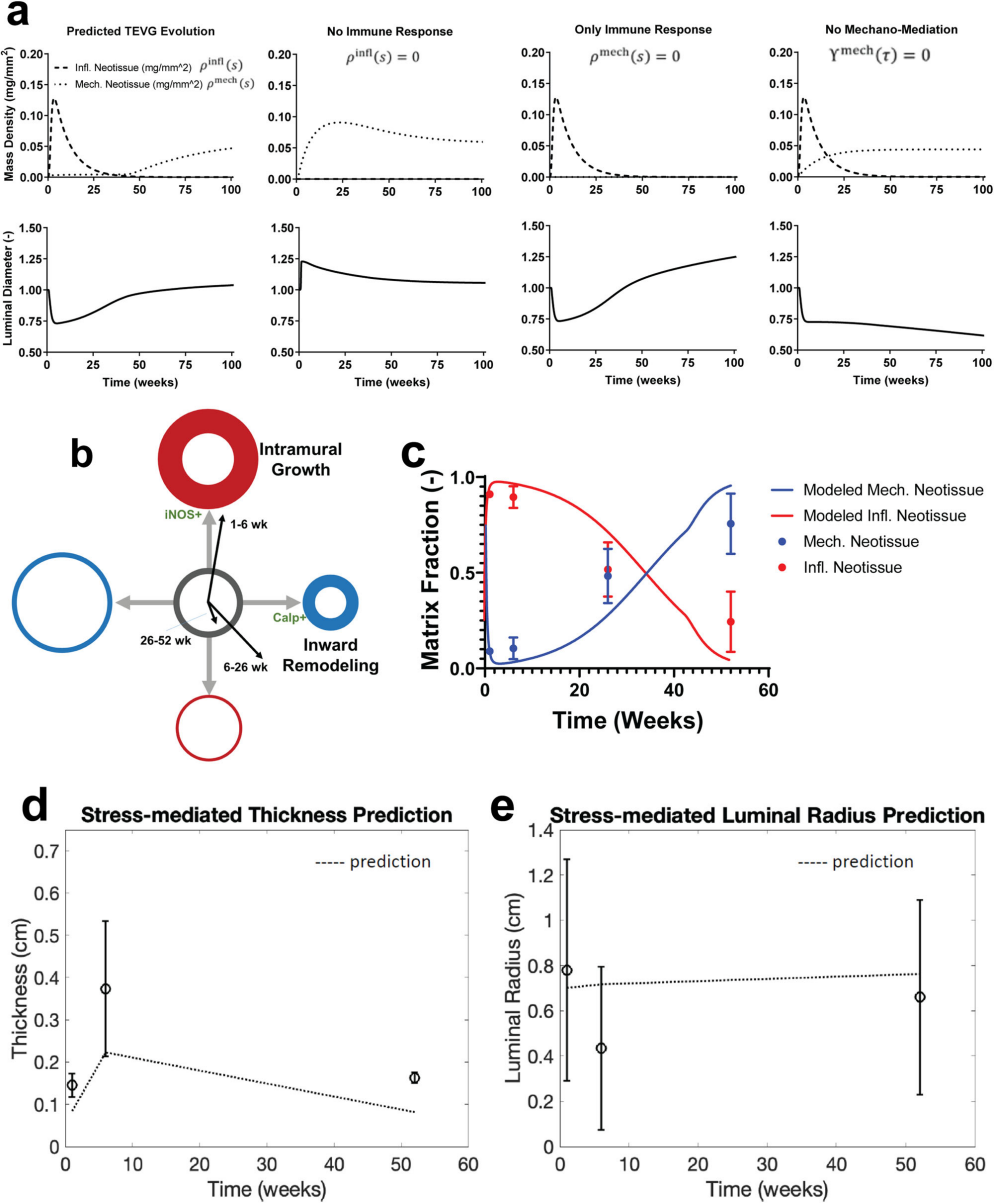
Computational model predicts TEVG neovessel formation. **A** Computational G&R modeling of TEVG neotissue formation demonstrating the combinatory effects of inflammation and mechano-mediated neotissue formation and remodeling predicted for the experimentally tested graft (left). Additional computational modeling results for theoretical cases with the absence of an immune response (mid left), only an immune response (mid right), and a lack of mechano-mediation for neotissue production (right). **B** Plot of inward remodeling vs intramural growth, with changes over time demonstrated by arrows from the origin. **C** Comparison of computational modeling prediction of inflammatory and mechanical neotissue to histological findings. **D** Stress-mediated thickness prediction and **(E)** stress-mediated luminal area prediction based on hemodynamic and morphology measurements taken from a computational hemodynamics study and the assumption that perturbations in flow and pressure are proportional to their homeostatic values. Perturbed shear and circumferential stress calculated using the proportionality constants and resulting thickness and radius changes inferred. 52-week native IVC values were taken to be the homeostatic values. Measured values +/- standard deviation are shown through circular markers. All values were calculated at 77% of the distance from the proximal to distal anastomosis, where stenosis routinely occurs in 6-week sheep. TEVG: tissue-engineered vascular graft, G&R: growth and remodeling, IVC: inferior vena cava.

Previous clinical studies and computational simulations demonstrated that the TEVGs were prone to early stenosis^11^, but simulations revealed a possible spontaneous reversal as the balance of an immuno-dominant response (${\rho }^{{{{{{\rm{infl}}}}}}}\gg {\rho }^{{{{{{\rm{mech}}}}}}}$, ${\Upsilon }^{{{{{{\rm{mech}}}}}}}\, < \,0$) shifted towards a mechano-mediated response (${\rho }^{{{{{{\rm{mech}}}}}}}\gg {\rho }^{{{{{{\rm{infl}}}}}}}$, ${\Upsilon }^{{{{{{\rm{mech}}}}}}}\, > \,0$) between 25 and 50 weeks with scaffold degradation and geometric changes (Fig. 7A). We probed the importance of these mechanisms in silico by isolating the effect of each stimulus. When the immune response to the scaffold was eliminated numerically (set ${\rho }^{{{{{{\rm{infl}}}}}}}(s)=0$), the simulated TEVG experienced an early-onset rapid dilation due to a lack of early neotissue formation as the scaffold degraded. Over time, however, mechano-mediated neotissue progressively restored the TEVG towards its original diameter, which was mediated to reach to homeostatic WSS. Conversely, including only immuno-mediated neotissue production (set ${\rho }^{{{{{{\rm{mech}}}}}}}(s)=0$) led to an early stenosis followed by reversal, but the lack of mechano-mediated neotissue production led to substantial dilatation of the graft at late times as the immuno-mediated neotissue degraded without new production to replace it. Finally, without mechano-mediation to reduce the production of native-like neotissue (set ${\Upsilon }^{{{{{{\rm{mech}}}}}}}(\tau )=0$) during the peak inflammatory response, the stenosed geometry was “locked-in”. These simulations highlight the relative importance of the extent and timing of both immuno- and mechano-mediated neotissue formation in TEVG behavior at different times during TEVG evolution.

From the G&R modeling results, we then quantified relative contributions of inflammation-driven and mechanical-mediated constituents from the predicted TEVG evolution on neotissue G&R over the simulated 52-week period, which confirmed that neotissue formation was driven primarily by inflammation during the first 26 weeks after implantation with a rapid transition to mechano-mediated neotissue formation thereafter (Fig. 7B).

Comparison of our computational G&R predictions of inflammatory versus mechanically stimulated neotissue production with explant histology (CD45 for inflammatory cells and calponin for mechanically stimulated neotissue) revealed good agreement. The relative amount of mechanical and inflammatory neotissues measured from histology was qualitatively similar at 26 weeks while the model predicted this similarity at approximately 36 weeks after implantation (Fig. 7C). In vivo measurements also demonstrated a longer-lasting inflammatory response than seen in the modeling predictions (Fig. 7C). Mechano-mediated growth predictions based on the FSI results and 3D anatomical models (Fig. 7D, E) further supported our timeline for inflammation-driven and mechano-mediated responses, with increasing inflammation-driven responses up to 6 weeks but modest at 52 weeks, when the mechano-mediated response accounts for most, but not all, of the in vivo thickness measurements. To test whether the long-term stress-mediated thickness and radius reflected homeostatic values, we evaluated the computational simulations using theoretical results inferred from native vessels^28^. That is, for fold-increases in flow and pressure (ε and γ, respectively) relative to original (homeostatic) values, mean WSS and intramural stress can be written as

$${\tau }_{w}=\frac{4\mu (\varepsilon {Q}_{0})}{\pi {r}^{3}},{\sigma }_{\theta }=\frac{\gamma {P}_{0}r}{h},$$

where *Q* is the volumetric flowrate, *P* the pressure, *r* the luminal radius, and *h* the wall thickness, with subscript 0 indicating homeostatic values. A return to homeostatic values of intramural stress and wall shear stress requires remodeled radius and thickness values to be $r={\varepsilon }^{1/3}{r}_{0},h={\varepsilon }^{1/3}\gamma {h}_{0}$. We took 52-week native IVC values to represent the homeostatic state. The difference between predicted (stress-mediated) and measured wall thickness thus represented the thickness contribution from the immune response. Similarly, the difference in predicted luminal radius from the mechano-adaptive case and the radial evolution measured experimentally demonstrate the effect of inflammation on vessel narrowing. The role of the immune response, i.e. the large difference in the mechano-adaptive prediction (dashed line, Fig. 7D, E) and the actual geometric evolutions (symbols, Fig. 7D, E) was apparent at 6 weeks but decreased dramatically by 52 weeks, further supporting that the immune response dominated early G&R before giving way to more mechano-mediated responses stimulated by the changing hemodynamics.

The relative contributions of, and relationships between, inflammation-driven and mechano-mediated G&R were demonstrated further by correlating our morphometric and IHC data over time. These data revealed a significant positive correlation between iNOS and intramural growth (linear regression *p* < 0.0001, *R*^2^=0.628), supporting the model-based prediction of inflammation-mediated luminal narrowing and experimental observations of inflammation-driven wall thickening. There was also a significant positive correlation between calponin and inward remodeling, although with a weaker R^2^ correlation coefficient (linear regression *p* = 0.0023, *R*^2^ = 0.278), supporting the model prediction of mediation of geometric changes by mechano-sensitive smooth muscle cells and our experimental data demonstrating the role of inward remodeling in stenosis around 6 weeks after implantation (Supplemental Fig. 4). Of note, a decrease in thickness of a pressurized tube while retaining identical material properties would have the effect of increasing the outer diameter through reduced structural stiffness. As the TEVG decreased in outer diameter and wall thickness, this suggests that a change in material stiffness is likely, as confirmed via biomechanical testing. Furthermore, eNOS staining for endothelial cells demonstrated little to no staining at 1 week, scattered staining along the lumens of the TEVGs at 6 weeks, and complete luminal staining at 26 weeks and beyond, similar to the staining along the lumen of the native IVC in appearance (Supplemental Fig. 5). Evolution of an intact endothelium corresponded with the cells’ potential ability to respond to mechanical stimuli exerted by WSS.

### Neovessels evolve to resemble native vessels in structure and function

**Fig. 8:**
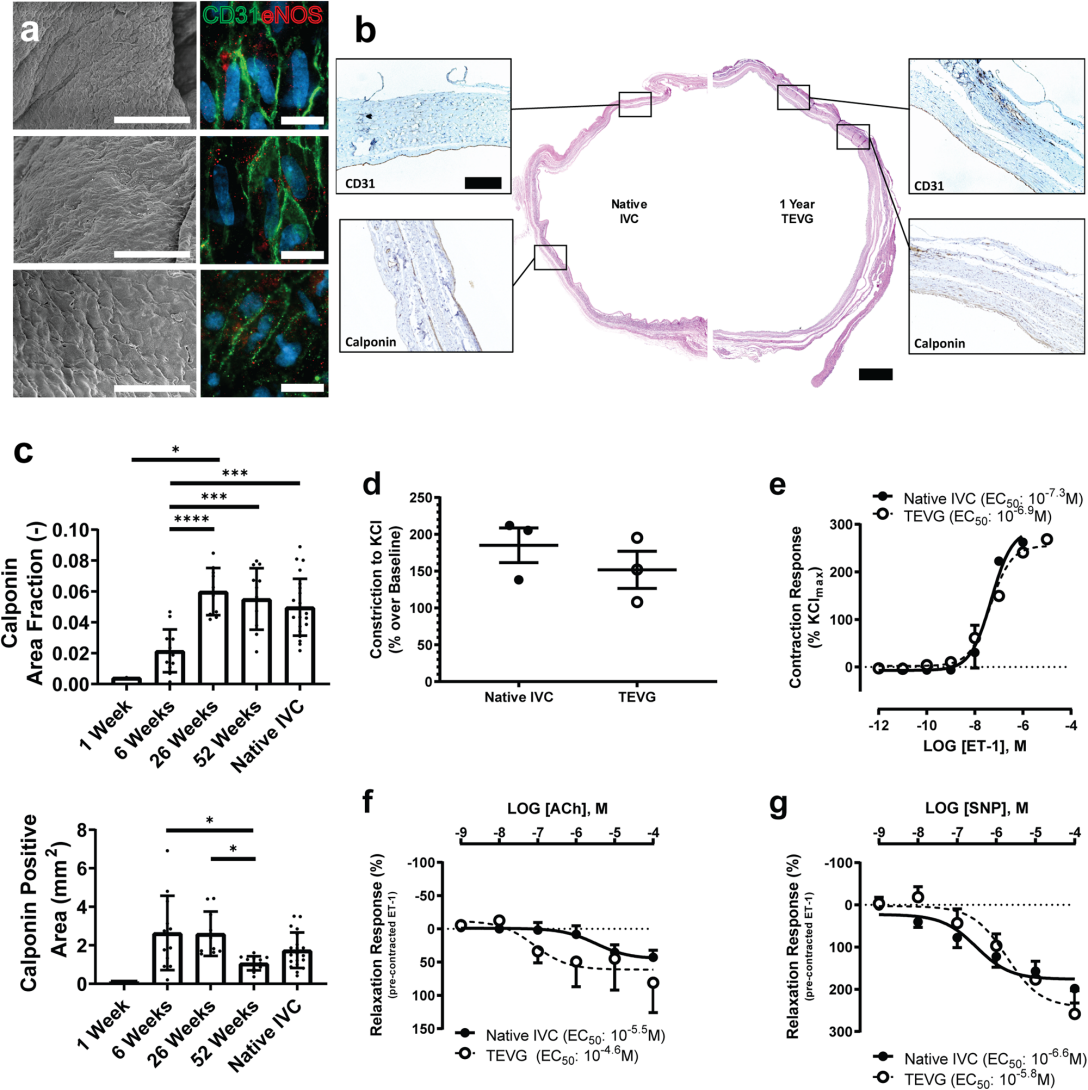
TEVGs develop into neovessels with native structure and vasoreactivity. **A** SEM (left) and *en face* immunofluorescent staining (right) of explanted TEVG neovessel luminal surface demonstrated confluent layer of endothelial cells; CD31 marked with green and eNOS with red. Scale bar left 50 µm, right 20 µm. **B** Representative H&E histology of native IVC (left) to 52-week TEVG (right), with insets showing CD31-lined lumen (top) and layers of calponin-positive smooth muscle cells (bottom). Scale bars H&E 1 mm, CD31 & calponin 200 µm. **C** Quantification of calponin staining from explanted TEVGs (*N* = 1, 12, 10, 12, 25 for 1 week, 6 weeks, 26 weeks, 52 weeks, and native respectively). Results of vasoreactivity testing of TEVGs (*N* = 3 for each) implanted for over 78 weeks and adjacent native IVC, demonstrating comparable responses to KCl (**D**), ET-1 (**E**), ACh (**F**), and SNP (**G**). Data shown as mean ± SD. Statistical significance determined using ANOVA with Tukey post-hoc test. *<0.05, **<0.01, ***<0.001, ****<0.0001. TEVG: tissue-engineered vascular grafts, SEM: scanning electron microscope, IVC: inferior vena cava.

Our computational G&R model suggested that after scaffold degradation, G&R of the TEVG would progressively yield a neovessel that mimics the native vessels. At 52 weeks post-implantation, surface SEM demonstrated a contiguous luminal covering of endothelial cells, and *en face* immunofluorescent staining of the luminal surface with CD31 and eNOS suggested functional endothelial cells, although not necessarily having fully native levels of functionality (Fig. 8A). IHC comparisons of the neovessel and the native vessel demonstrated that the 52-week old neovessel had a thin laminated wall composed of layers similar to those seen in the native IVC (Fig. 8B). The intima was composed of a monolayer of CD31+ endothelial cells surrounded by concentric layers of calponin+ smooth muscle cells. Time course IHC studies revealed further that calponin+ smooth muscle cells increased rapidly from 6 to 26 weeks (calponin+ area fraction was 0.021 ± 0.014 at 6 weeks vs 0.060 ± 0.015 at 26 weeks, t-test *p* < 0.001), after which the levels remained similar to those seen in the native IVC (Fig. 8C: calponin+ area fraction of 0.055 ± 0.020 for the 52-week TEVG vs 0.050 ± 0.019 for the IVC, t-test *p* = 0.485). When examining the total amount of calponin+ area, it increased from 1 to 6 weeks, then stayed steady until 26 weeks before declining at 52 weeks as the neovessel matured.

To further characterize neovessel functionality, we subjected three long-term implants (>1.5 years) to vasoreactivity testing (Fig. 8D–G). Results demonstrated that the neovessels had similar contractile responses (as measured by force in a ring myograph) to that of the native IVCs in response to both potassium chloride (KCl) and endothelin-1 (ET-1) stimulation (Fig. 8D, E). The neovessels also vasodilated (as measured by force) similar to the native IVCs in response to acetylcholine (ACh) (Fig. 8F), an endothelial dependent generator of eNOS/NO, and sodium nitroprusside (SNP), an endothelial-independent NO donor (Fig. 8G**)**. Together with the histological findings, resolution of inflammation, comparison of wall shear stress measurements, and the observation that the implanted scaffold is composed of polymer and therefore pharmacologically inert at implant, these results suggested the development of native-like structure and function.

### Neovessels exhibit biological growth

**Fig. 9:**
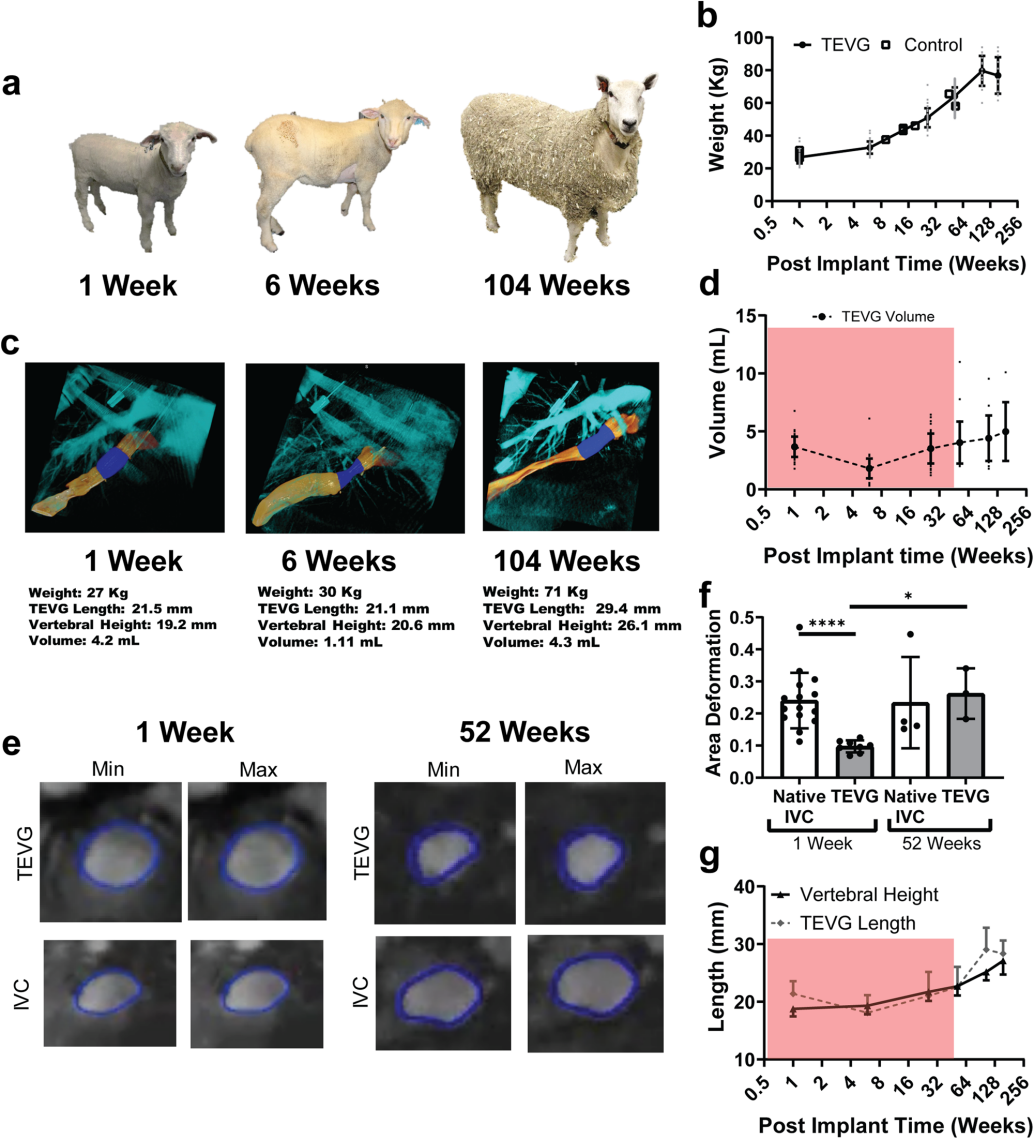
TEVG neovessels demonstrate biological growth. **A** Representative images of growth of sheep over implantation time. **B** Quantification of weight for TEVG-implanted and non-implanted control animals over long-term implantation. **C** Representative 3D angiography imaging of a sheep over the implantation time. Native IVC colored yellow, TEVG colored dark blue, and surrounding anatomic structures colored light blue. Measurements taken from each representative image shown below. **D** Quantification of TEVG volume over time. **E** Representative images of mid-graft TEVG at minimum and maximum area over a cardiac cycle as measured by MRI, at 1-week (Left) and 52-week (Right). **F** Quantification of area deformation of TEVG and adjacent IVC at 1-week and 52-week post-implantation. **G** Length of TEVG and vertebral body as measured from angiography. Red boxes denote the time until complete TEVG degradation. Data shown as mean+/-SD. Statistical significance in area deformation data determined using Mann-Whitney test for unequal variances test. *<0.05, **<0.01, ***<0.001, ****<0.0001. TEVG: tissue engineered vascular grafts, IVC: inferior vena cava, MRI: magnetic resonance imaging.

In addition to investigating neotissue deposition and remodeling into functional neovessels, we sought to evaluate the biological growth potential of the TEVG. Biological growth refers to the progressive change in size, shape, and function that occurs during the development and maturation of an organism. Because we implanted the TEVG in juvenile (4-month old) lambs, we were able to evaluate the biological growth potential of the neovessels as the lambs matured to adult sheep (Fig. 9A). The lambs more than doubled in body mass during the first year following implantation (26.8 ± 3.8 kg at 1 week, 64.2 ± 5.5 kg at 52 weeks, t-test *p* < 0.001) and continued to grow steadily out to two years before leveling off (79.6 ± 9.2 kg at 104 weeks, 76.8 ± 11.2 kg at 156 weeks, t-test *p* = 0.446) (Fig. 9B). Comparing our implanted animals and age-matched non-implanted controls revealed similar growth, suggesting that the TEVG implant in the IVC did not cause any growth restriction of the animal.

Volumetric reconstructions based on serial 3D angiography of the TEVG demonstrated that the TEVG lumen initially decreased in volume, reaching its nadir at 6 weeks after implantation (3.6 ± 0.9 mL at 1 week vs 1.8 ± 0.9 mL at 6 weeks, t-test *p* < 0.001), then increased in volume over the ensuing 150-week period (3.5 ± 1.3 mL at 26 weeks vs 5.0 ± 2.5 mL at 156 weeks, t-test *p* = 0.018) (Fig. 9C, D). During the same period, the TEVG became progressively more compliant. Comparison of the luminal area deformation of the TEVG and IVC over the cardiac cycle by MRI revealed that the TEVG was relatively stiff upon implantation compared to the IVC at 1 week (0.24 ± 0.08 IVC vs 0.10 ± 0.02 TEVG, fractional area deformation, Mann-Whitney test *p* < 0.0001), but by 52 weeks post-implantation the neovessel appeared to pulse similarly to the surrounding native IVC (0.23 ± 0.12 IVC vs 0.26 ± 0.06 TEVG, fractional area deformation, Mann-Whitney test *p* = 0.400) (Fig. 9E, F). This increase in compliance was important because the IVC is a highly compliant vessel that changes its volume dramatically based on the hemodynamic forces, which allows it to function as a capacitance vessel. Yet, assessing growth based on volume or diameter alone could be confounded by differences in the hemodynamic states of an animal at different ages. In contrast, the length of a vessel was not affected by the hemodynamic state and therefore represented a better measure of biological growth capacity. Thus, we measured the change in TEVG length over time and compared it to the change in vertebral body height measured on the same angiogram. Serial measurements revealed that the TEVG initially decreased in length during the first 6 weeks (21.4 ± 2.2 mm at 1 week, 18.1 ± 3.1 mm at 6 weeks, t-test *p* = 0.001) then subsequently increased in length (21.0 ± 4.1 mm at 26 weeks vs 28.3 ± 2.3 mm at 156 weeks, t-test *p* < 0.001) at a rate similar to the rate of change in the vertebral body, which coincidentally had a length similar to that of the implanted TEVG (21.7 ± 1.6 mm at 26 weeks vs 27.12 ± 2.4 mm at 156 weeks) over the ensuing time course (Fig. 9G).

## Discussion

We used an integrative computational-experimental approach to quantify the natural history of G&R in TEVGs implanted in the ovine venous circulation. We used two separate computational frameworks: (i) a one-dimensional G&R framework that accounts for evolving changes in geometry (diameter and thickness), composition, and material properties due to immuno- and mechano-mediated stimuli and (ii) a three-dimensional FSI framework which accounts for complex hemodynamics within subject-specific geometries. G&R were captured using a constrained mixture model^11,26^, parameterized previously, that accounts for different natural configurations, material properties, and mass fractions of different structurally significant constituents, accounting for an evolving TEVG that consists of changing fractions of polymer and oriented ECM and cells. This model highlighted the critical role the scaffold plays in inducing both inflammation-driven neotissue formation and mechanically-mediated remodeling (Fig. 7A). In silico experiments that removed mechano-mediation of neotissue production and removed the long-term presence of native-like neotissue suggested a role for mechanics in guiding the development of a neovessel that behaves as native vessel. For these cases without native-like mechanobiological responses, long-term narrowing and dilatation were predicted, respectively. Therefore, stabilization of the luminal area after reversal of stenosis (Fig. 2B) suggested that the inclusion of mechanosensitivity of the neotissue was important for understanding the long-term evolution. These G&R-based predictions suggested further that we evaluate how the inflammation-inducing and stress-shielding characteristics of the biodegradable scaffold evolve, induce, and modulate neotissue formation in vivo. In particular, results of our data-informed computational model suggested critical dynamic changes during the first 26 weeks after TEVG implantation, during which the scaffold largely degrades, and cell/matrix turnover following scaffold degradation resulting in a neovessel that can grow and remodel similar to the native vessels. These findings were further supported by results of the FSI simulations, which revealed low correlation values between hemodynamics and morphological changes over the first 6 weeks, when inflammation is dominant, but progression to homeostatic values at later times when mechanically-mediated remodeling is dominant.

These findings compared well with trends seen in our experimental degradation and histological data, which suggested that the first 6 weeks of neotissue formation were driven by inflammation. This resulted in luminal narrowing due to a thickened wall composed primarily of inflammatory neotissue within the scaffold. Between 6 and 26 weeks, the degree of inflammation-driven neotissue formation decreased as the scaffold degraded, resulting in wall thinning and an increasing lumen diameter. These changes were partially offset, however, by the initiation of mechano-mediated remodeling as the scaffold became more compliant and lost its stress-shielding capacity, resulting in inward remodeling. Between 26 and 52 weeks, as the scaffold finished degrading, inflammation-driven neotissue formation continued to diminish, resulting in further wall thinning that was augmented by the mechano-mediated G&R as the ECM matured and the neovessel wall became more compliant and the lumen expanded while the wall thinned.

We previously demonstrated that cell seeding was not essential for neovessel formation, though it modulated outcomes^45–47^. In particular, the cells seeded onto the TEVG scaffold disappeared shortly after implantation and did not directly give rise to the vascular neotissue, as has been noted for many stem cell-related therapies in recent years^48–51^. Rather, the inflammation that was induced by the implanted polymeric scaffold was essential for neotissue formation^52–54^. Depletion of monocytes and macrophages using either clodronate liposomes or diphtheria toxin (using a transgenic DT CD11b mouse model) blocked vascular neotissue formation^53^. Results of cell tracking and cell lineage tracing experiments demonstrated that the endothelial cells and smooth muscle cells that gave rise to the intimal and medial layers of the neovessel arose from the neighboring vessel wall^55^. Thus, the monocytes and macrophages that infiltrated the scaffold induced the ingrowth of endothelial cells and smooth muscle cells along the surface of the scaffold via IL-10 and MCP-1 dependent paracrine signaling mechanisms^56^. We have previously shown that the phenotype of the infiltrating macrophages plays a critical role in neotissue formation^52,57,58^. Interestingly, the macrophages that infiltrated the scaffold exhibited both pro-inflammatory and anti-inflammatory markers, and the relative numbers of these cells remained constant throughout neovessel formation (Fig. 4B). This inflammatory-driven process was reminiscent of what occurs in lower order species that possess the ability to regenerate in that the regenerative process could be blocked by clodronate liposomes and the inflammatory cells simultaneously exhibited both pro- and anti-inflammatory phenotypes that drove the regenerative process^59–61^. In other vascular applications, such as vascular remodeling after stent placement, examination of these mechanisms may be key to understanding the potential for reversal of intimal hyperplasia^62–64^.

While inflammation is essential for neovessel formation, excessive inflammation leads to TEVG stenosis. Results of the current study demonstrated that, in the sheep IVC interposition graft model, inflammation reached its highest measured levels at around 6 weeks and decreased thereafter as the scaffold degraded. The bulk of the wall thickening leading to the early TEVG stenosis was driven by inflammatory cells as the scaffold area increased during the degradation process. Subsequent wall thinning occurred between 6 and 26 weeks after implantation due to resolution of the foreign body reaction, as scaffold material was degraded and resorbed.

Previously, we postulated that late stage G&R experienced in our clinical studies was flow-dependent^65^. Both computational models provided further support of this hypothesis. A central hypothesis of a mechano-mediated G&R model is that blood vessels actively work to maintain a desired homeostatic state. This response requires coordinated changes in luminal radius and wall thickness based on fold-changes in hemodynamics from baseline^3,28^. When considering a 1-year-old native IVC as the desired homeostatic state **(**Fig. 7D, E**)**, the critical role of the immune response was apparent in the TEVG at 6 weeks as wall shear stress was inhomogeneous across the TEVG length (Fig. 8D) but decreased dramatically by 52 weeks with the wall shear stress becoming more homogeneous as it became more similar to that of the adjacent vessels. These results are consistent with our findings that the immune response dominated early G&R before giving way to a more mechano-mediated (i.e. hemodynamic-driven) response. Further, while the FSI simulation results demonstrate large changes in both pressure and WSS from 1 to 6 weeks during stenosis formation, these are not strongly correlated with local changes in TEVG geometry during this period. The endothelium was not intact until between 6 and 26 weeks, highlighting the improbability of WSS being sensed by endothelial cells prior to this time. Contrary to adaptive responses in normal mature vessels, where blood pressure is a major stimulus or driver of changes in wall thickness^66^, early TEVG neotissue formation was driven by inflammation, resulting in morphological changes up to at least 6-week post-implantation that were detrimental; thereafter, inflammation began to wane as the polymer degraded, and the mechanical stimuli could be increasingly sensed by cells since the polymer no longer stress-shielded them^67–69^. By 52 weeks, G&R of the TEVG was largely, though not entirely, mechano-mediated. The resulting TEVG remodeled to reach a similar level of WSS as the adjacent IVC, in accordance with vascular remodeling paradigms^70,71^.

Including terms to describe mechano-mediated G&R in the TEVG simulations was essential for accurately describing and predicting the in vivo observations and associated histology in our experimental studies. During the first 6 weeks after implantation, the scaffold provided significant stress-shielding due to the high stiffness of the TEVG. Thereafter, this stress-shielding effect of the scaffold diminished due to scaffold degradation. Scaffold mechanics have been previously shown to have large effects on neotissue formation in vivo^72–74^. The present study emphasized the increasing role of the cellular mechanobiology as the scaffold degraded and its stress-shielding properties diminished. Beyond 26 weeks after implantation, when the scaffold lost its stress-shielding properties, G&R was mainly mechano-driven and appeared to be well described in terms of mechanical homeostasis with intramural stresses in both the circumferential and axial directions not significantly different than the native values (Fig. 5B). During this period, the ECM remodeled to resemble the compliance and stress state of the native IVC (Fig. 2) and became vasoreactive (Fig. 8D–F). It should be noted, however, that the vasoreactivity studies performed here included only three samples, and included multiple factors that could function independent of endothelial cell functionality. As eNOS is released in response to WSS as well as inflammation, and eNOS can be uncoupled from stimulation resulting in reactive oxygen species production, these findings should be considered preliminary and will require further evaluation in future studies. Further testing with more samples, a wider array of stimulatory molecules, and more time points will provide valuable information about the timeline and degree of development of neovessel vasoreactivity.

The computational models shed light on the biological growth potential of the TEVG. During the first phase, the period of neotissue formation, the polymeric scaffold prevented normal biologic growth. This was particularly noticeable during the first 6 weeks after implantation when neotissue formation was primarily inflammation-driven due to the presence of the scaffold, which restricted growth and decreased the length of the TEVG while native vertebral height increased (Fig. 9G). After 6 weeks, when the scaffold lost its biomechanical integrity due to polymer degradation, biological growth was no longer restricted and the TEVG grew in length as it was loaded axially by the growing thoracic cavity, again suggesting mechanosensitivity to the loading environment to allow normal vascular G&R. Yet, residual inflammatory scaffold material continued to affect G&R until it was substantially degraded by 26 weeks. Once the scaffold was completely degraded, after 52 weeks (Fig. 9), the neovessel possessed near normal biological growth capacity. In other words, neovessels, not TEVGs, possess growth potential.

An important benefit to our computational-experimental approach is the ability to guide future experimentation via time- and cost-efficient simulations as well as the ability of experimental results to inform and refine the computational model. As the composition and mechanical properties of the scaffold were guiding forces of inflammation and neotissue formation during the early period, computational modeling could be beneficial in navigating the potential scaffold parameter space in silico^43^. Coupled with guided in vivo experimentation around the edges of this parameter space, this computational-experimental approach can suggest key times and scaffolding parameters for empirical evaluation while experimental results can be compared and contrasted with modeling outcomes to determine new effects and interactions to create a more accurate model^72,75,76^. Recently, the Hoerstrup group has had similar success in combining computational modeling with in vivo experimentation of tissue-engineered heart valves in sheep to determine performance^77^. This method has the further benefit of decreasing the number of animals needed for future experimentation, and may accelerate new design development by suggesting optimized scaffold parameters. 3D FSI modeling helped us to understand asymmetrical remodeling through mapping the entire region of interest. It may also enable the evaluation of regions that are particularly sensitive to hotspots or spikes in hemodynamic forces. Ultimately, 3D models will help us understand how the geometry affects the hemodynamically induced loading, noting that the loading drives changes in composition that affect the TEVG geometry, and so forth.

This study identified several characteristics of the scaffold that could potentially be modified or optimized to improve TEVG performance. First, differing timelines of degradation of the immuno- and mechano-mediated properties of the scaffold resulted in early TEVG narrowing; modifying these relative timelines could lead to improved performance. However, it is worth noting that this early narrowing was shown to not reach clinically significant levels in clinical trial TEVG patients^78^. Second, the sudden loss of stress-shielding as the scaffold transformed from relatively stiff to highly compliant arose from the breaking of degrading polymer fibers resulting in loss of structural integrity. A more gradual transition could minimize the dynamic changes in G&R exhibited during the first 26 weeks after implantation, a concept that has shown benefit in related G&R applications^79^. Third, the scaffold degrading through hydrolysis allowed analysis of degradation in vitro. Yet, our results revealed differences in in vivo degradation rates that manifested as heterogeneous distributions of small amounts of residual polymer in specimens implanted beyond 26 weeks. Though limited in amount, these materials served as a persistent stimulus of inflammation and possible local stress-shielding, both of which could impact G&R. While the in vitro degradation process was accomplished through a purely chemical process, the in vivo degradation is modified by a number of factors, including inflammatory cytokines and long-term inflammation, fibrosing encapsulation of polymer materials and giant cell formation, and the effects of changing mechanical and hemodynamic loads upon the degrading scaffold. These factors have multiple accelerating and decelerating effects, resulting in differences in the local rate of polymer degradation. The result is a process that, at the bulk level, appears to correlate well between in vitro and in vivo studies, while at the cellular level can create areas where the local degradation rate and inflammatory stimulus is higher or lower than predicted. More homogenous and complete degradation in vivo could lead to more native-like remodeling during the neotissue formation and neovessel remodeling phases.

One limitation of our current computational G&R model was that it was not designed to describe or predict individual graft performance, but instead to describe the mean population behavior. Thus, G&R for any individual TEVG may deviate from the model-based predictions. By contrast, the FSI simulation incorporated subject-specific geometry and boundary conditions. Moving forward, our predictive capabilities would benefit from a fluid-solid growth (FSG) model that melds current FSI and G&R models, enabling individualized predictions. Similarly, recent work has used targeted molecular imaging to quantify individual foreign body reactions to the TEVG scaffold, which could be employed to improve the model’s ability to describe and predict the in vivo behavior of individual TEVGs, opening the door to a novel personalized medicine approach for managing patients receiving a TEVG^80^. Future FSI models could also be improved by accounting for the “atrial kick”, which plays a role in the complex hemodynamics of the IVC. This could be accomplished using a lumped parameter heart model as well as boundary conditions that allow longitudinal extension of the vessel.

Autologous biological vascular conduits significantly outperform synthetic or other biological grafts^81^. Unfortunately, autologous vascular tissue for performing major cardiovascular reconstructive procedures is limited, necessitating the use of synthetic or non-autologous biomaterials for most major congenital heart operations. The use of these biomaterials contributes to significant morbidity and mortality. The fundamental premise underlying the development of a TEVG is that it would increase the supply of autologous vascular tissue for surgical reconstruction. Herein, we demonstrated that the TEVG transformed into a neovessel that eventually behaved like a native vessel, though with caveats of adverse G&R during the first 6 months after implantation. Nevertheless, following this critical period, the evolved neovessel possessed biological growth potential, with attributes of a native vessel, including a functional endothelial layer and a highly compliant and vasoactive wall that match well those of the vessel into which it is implanted. The development of these biomimetic properties has important implications for optimizing long-term graft performance, especially when used in the pediatric population. Continued utilization of a computational-experimental approach holds great promise for uncovering mechanisms underlying neovessel formation, which in turn can be used to direct their use, optimize their design, and improve their performance in a time- and cost-efficient manner.

### Reporting summary

Further information on research design is available in the Nature Research Reporting Summary linked to this article.

## Supplementary information

Supplemental Information

Supplementary Data 1

Supplementary Data 2

Supplementary Video 1

Supplementary Video 2

Reporting Summary

Description of Additional Supplementary Files

## Data Availability

Source numerical data for the main figures in the manuscript is available as Supplemental Data 1. Raw microscopy images and all other raw datasets are available on reasonable request to the corresponding author.

## Code availability

The computational model and code with detailed methods, including the theoretical background can be found in previous manuscript^11^.
