# Supplementary material for: Tissue engineered vascular grafts transform into autologous neovessels capable of native function and growth
Source: Commun Med (Lond). 2022 Jan 10;2:3. doi: 10.1038/s43856-021-00063-7 (PMC9053249; doi:10.1038/s43856-021-00063-7)

Supplemental Information

- Supplemental Table 1 – List of IHC Antibodies
- Supplemental Figure 1 – IVUS Data Linear Correlations
- Supplemental Figure 2 – Calibration for Degradation Study
- Supplemental Figure 3 – Summary of Morphological and Hemodynamic Indices (Mid-Graft)
- Supplemental Figure 4 – Histomorphometry Statistical Regressions
- Supplemental Figure 5 – eNOS-Positive Endothelium
- Supplemental Video 1 – Angio at 1 Week, 6 Weeks, 26 Weeks, 52 Weeks, 104 Weeks
- Supplemental Video 2 – MRI at 1 Week and 52 Weeks

Supplemental Table 1: IHC Antibodies

**Primary Antibody**	**Manufacturer**	**Catalog Number**	**Dilution**	**Antigen Retrieval Buffer**	**Host Species**	**DAB Time (min)**
MPO	Abcam	ab208670	1:4000	Citrate (pH 6.0)	Rabbit	10
CD68	Abcam	ab125212	1:1000	Citrate (pH 6.0)	Rabbit	10
CD64	Abcam	ab140779	1:10000	Citrate (pH 6.0)	Mouse	10
CD3	Abcam	ab16669	1:10000	Tris-EDTA (pH 9.0)	Rabbit	10
CD45	Abcam	ab10558	1:4000	Tris-EDTA (pH 9.0)	Rabbit	10
a-SMA	Dako	M0851	1:2000	Citrate (pH 6.0)	Mouse	2
Calponin	Abcam	ab46794	1:1500	Tris-EDTA (pH 9.0)	Rabbit	10
Ki67	Abcam	ab15580	1:5000	Citrate (pH 6.0)	Rabbit	10
Desmin	Abcam	ab1520	1:1000	Tris-EDTA (pH 9.0)	Rabbit	10
iNOS	Abcam	ab15323	1:750	Citrate (pH 6.0)	Rabbit	10
CD163	Abcam	ab182422	1:10000	Tris-EDTA (pH 9.0)	Rabbit	10
eNOS	Abcam	ab76198	1:10000	Citrate (pH 6.0)	Rabbit	10

Supplementary Figure 1: IVUS Data Linear correlations

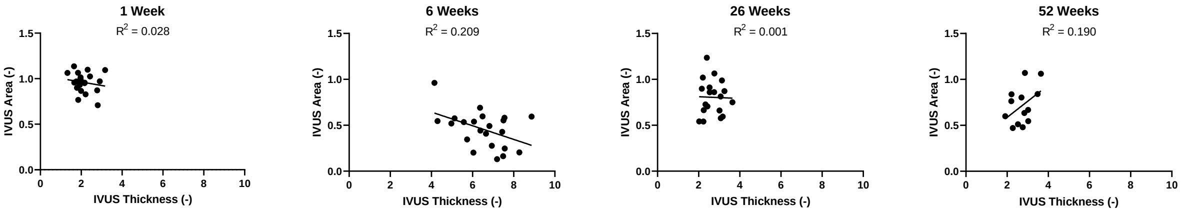

Supplemental Figure 2: Accelerated Degradation Study Calibration

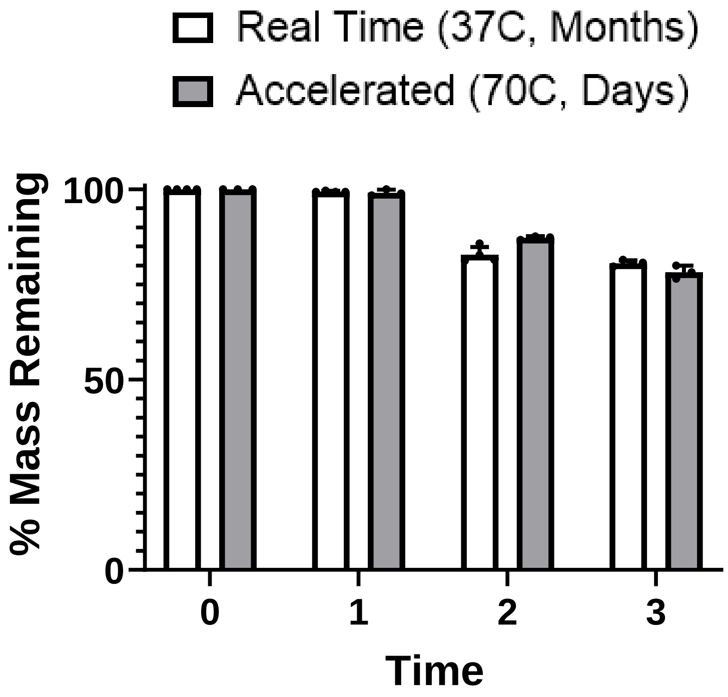

Supplemental Figure 3 – Summary of Morphological and Hemodynamic Indices (Mid-Graft)

mean			
time	1	6	52
area [cm^2]	2.24	1.18	1.56
pressure [mmHg]	1.97	9.62	2.09
thickness [cm]	0.15	0.25	0.17
volumetric flow [cm^3/s]	37.20	39.71	47.79
average velocity [cm/s]	18.02	37.82	34.71
cauchy stress invariant 1 [kPa]	1.51	18.69	1.46
TAWSS [dynes/cm^2]	16.35	19.04	42.24

std			
time	1	6	52
area	0.69	0.52	0.61
pressure	1.16	3.30	0.69
thickness	0.02	0.06	0.01
volumetric flow	4.46	13.62	5.72
average velocity	5.87	15.31	15.33
cauchy stress invariant 1	1.20	8.71	0.67
TAWSS	5.05	9.31	9.83

Supplemental Figure 4: Histomorphometry Statistical Regressions

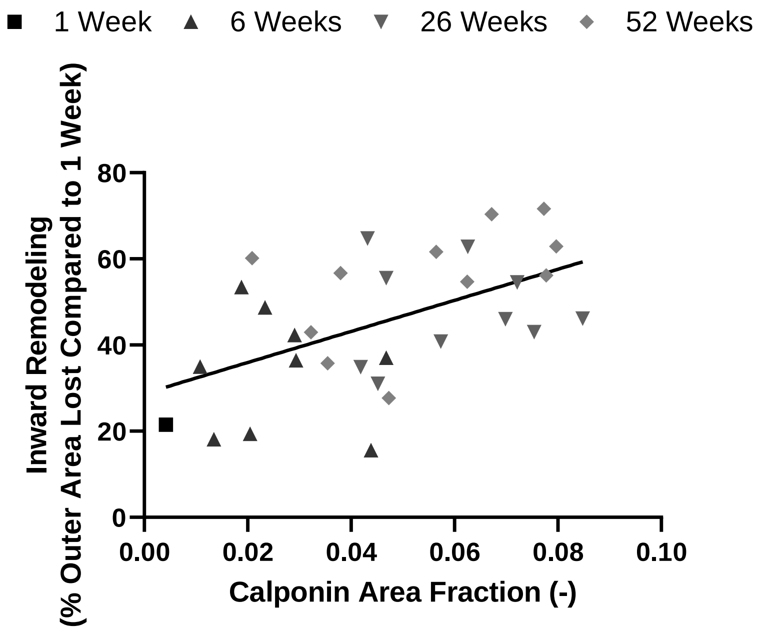

**Best-fit values**	
Slope	360.7
Y-intercept	28.73
X-intercept	-0.07964
1/slope	0.002772
**Std. Error**	
Slope	108.1
Y-intercept	5.554
**95% Confidence Intervals**	
Slope	139.6 to 581.8
Y-intercept	17.37 to 40.09
X-intercept	-0.2802 to -0.03059
**Goodness of Fit**	
R square	0.2775
Sy.x	13.47
**Is slope significantly non-zero?**	
F	11.14
DFn, DFd	1, 29
P value	0.0023
Deviation from zero?	Significant
**Equation**	Y = 360.7*X + 28.73
**Data**	
Number of X values	35
Maximum number of Y replicates	1
Total number of values	31
Number of missing values	4

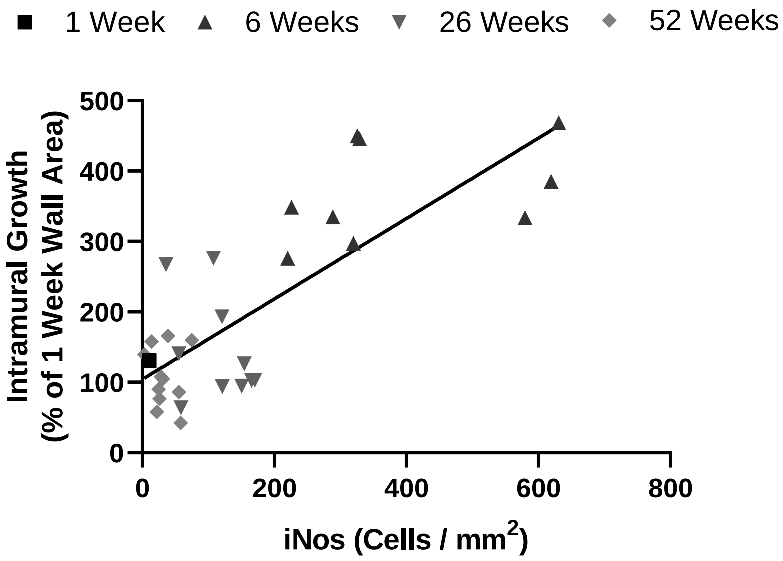

Best-fit values	
Slope	0.5708
Y-intercept	104.2
X-intercept	-182.5
1/slope	1.752
**Std. Error**	
Slope	0.08158
Y-intercept	19.59
**95% Confidence Intervals**	
Slope	0.4040 to 0.7377
Y-intercept	64.13 to 144.2
X-intercept	-337.8 to -91.90
**Goodness of Fit**	
R square	0.6280
Sy.x	79.89
**Is slope significantly non-zero?**	
F	48.96
DFn, DFd	1, 29
P value	<0.0001
Deviation from zero?	Significant
**Equation**	Y = 0.5708*X + 104.2
**Data**	
Number of X values	35
Maximum number of Y replicates	1
Total number of values	31
Number of missing values	4

Supplemental Figure 5: eNOS-Positive Endothelium

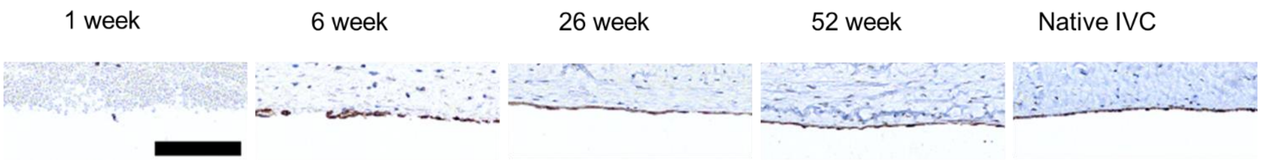

Supplement: Supplementary file 1 — Supplemental Information [file 43856_2021_63_MOESM1_ESM.pdf]
