# Supplementary material for: Tissue engineered vascular grafts transform into autologous neovessels capable of native function and growth
Source: Commun Med (Lond). 2022 Jan 10;2:3. doi: 10.1038/s43856-021-00063-7 (PMC9053249; doi:10.1038/s43856-021-00063-7)
Supplement: Supplementary file 4 — Supplementary Video 1 [file 43856_2021_63_MOESM4_ESM.pptx]

## Slide 1
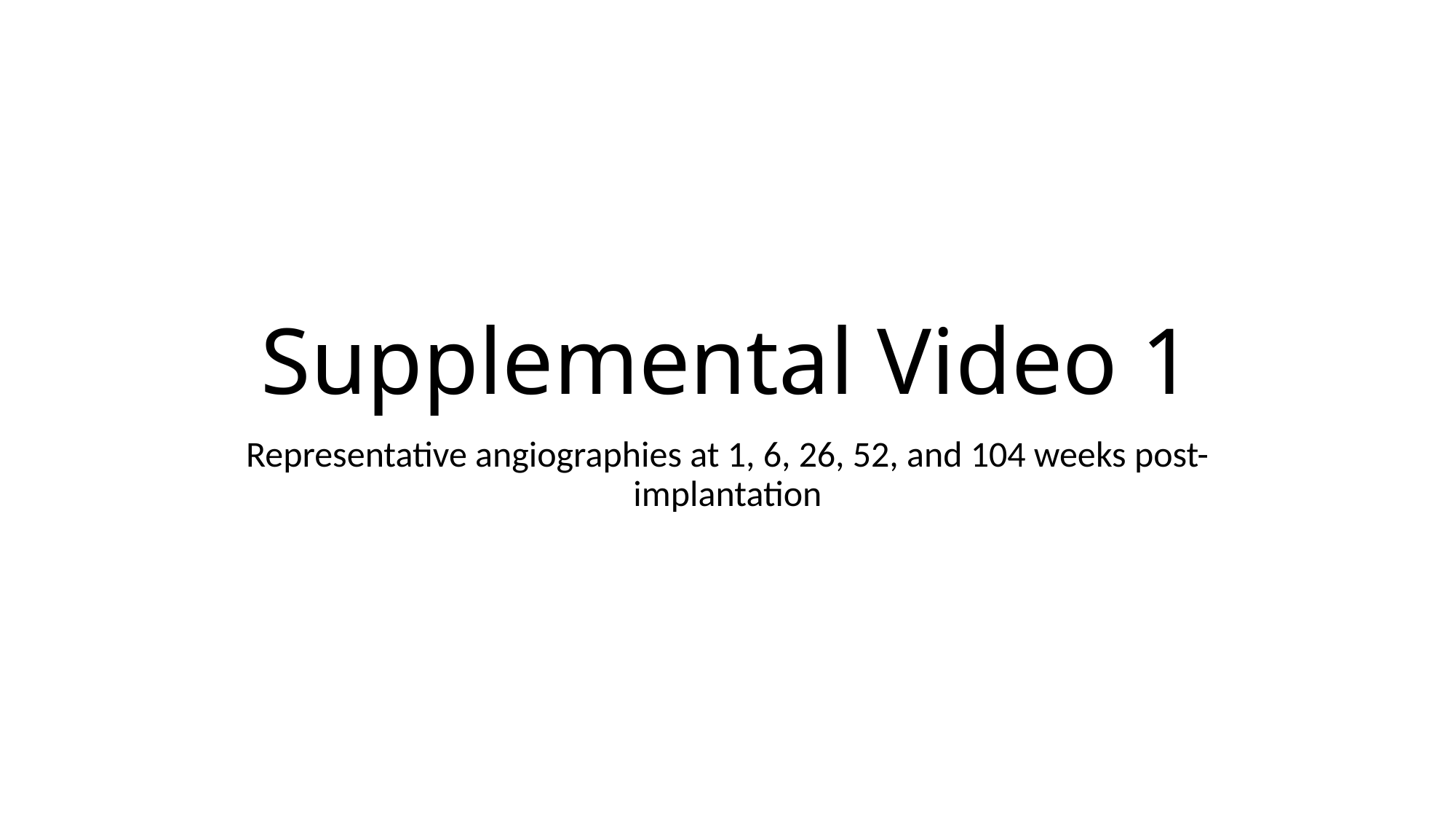

# Supplemental Video 1
Representative angiographies at 1, 6, 26, 52, and 104 weeks post-implantation

## Slide 2
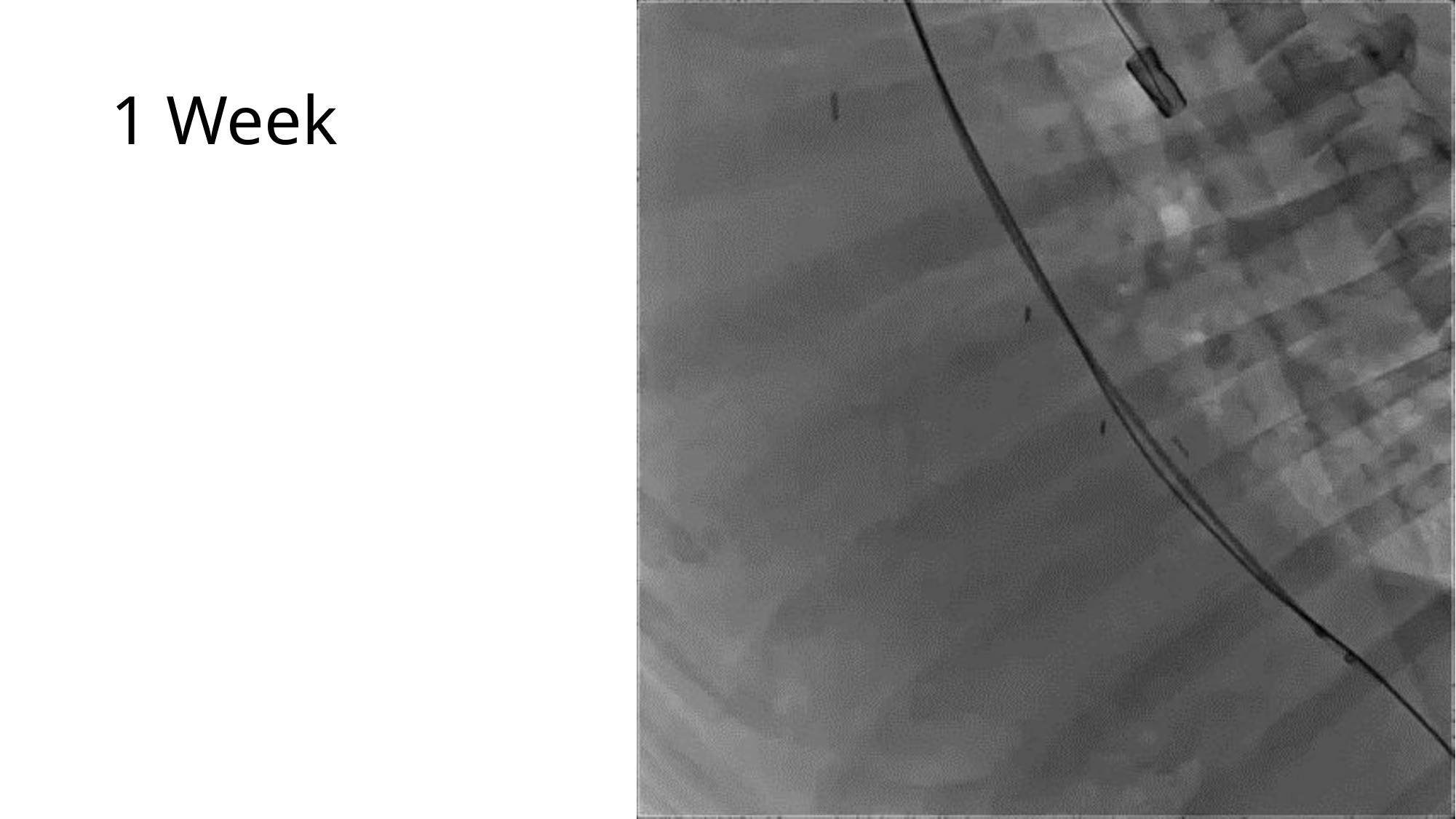

# 1 Week

## Slide 3
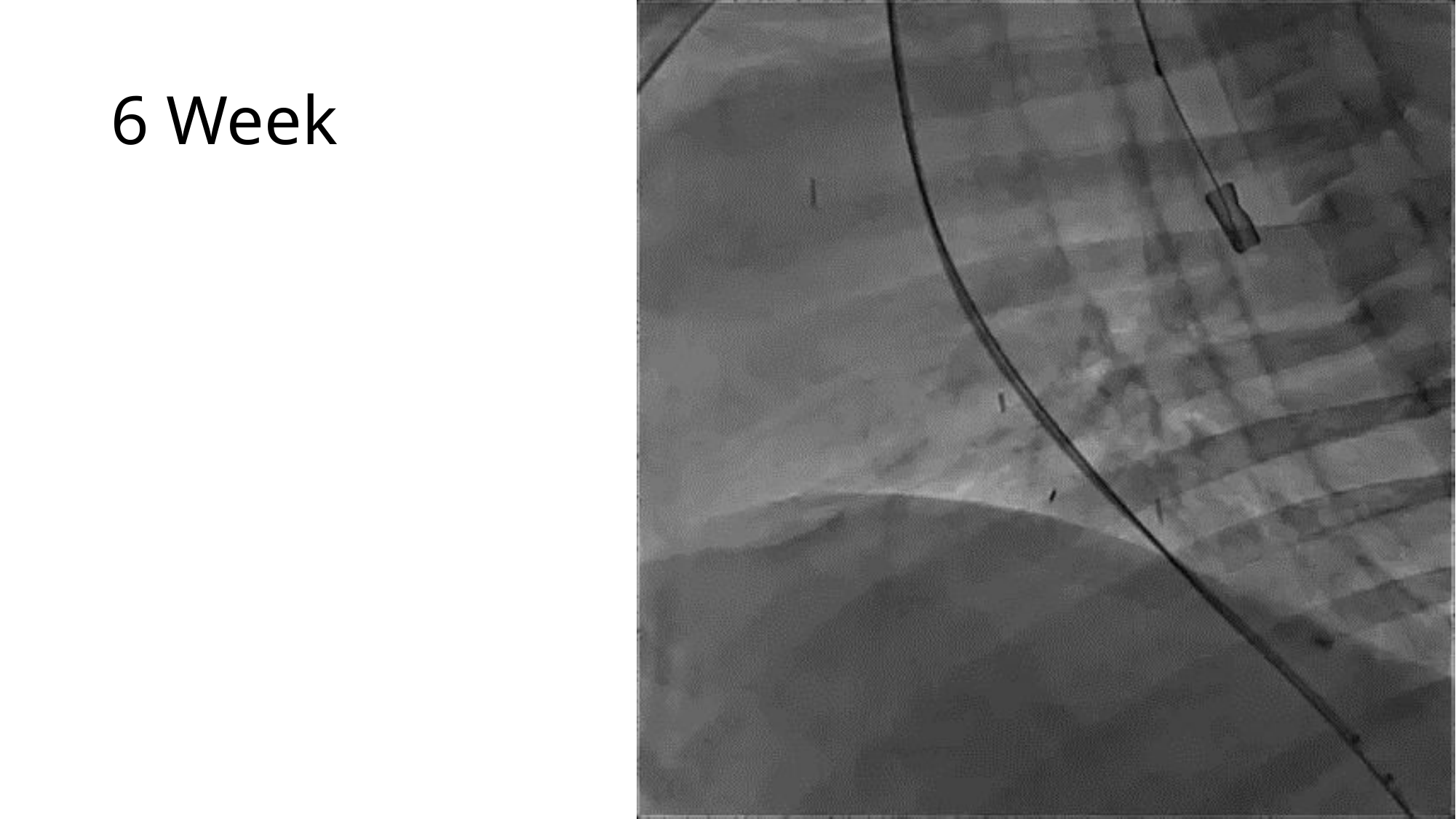

# 6 Week

## Slide 4
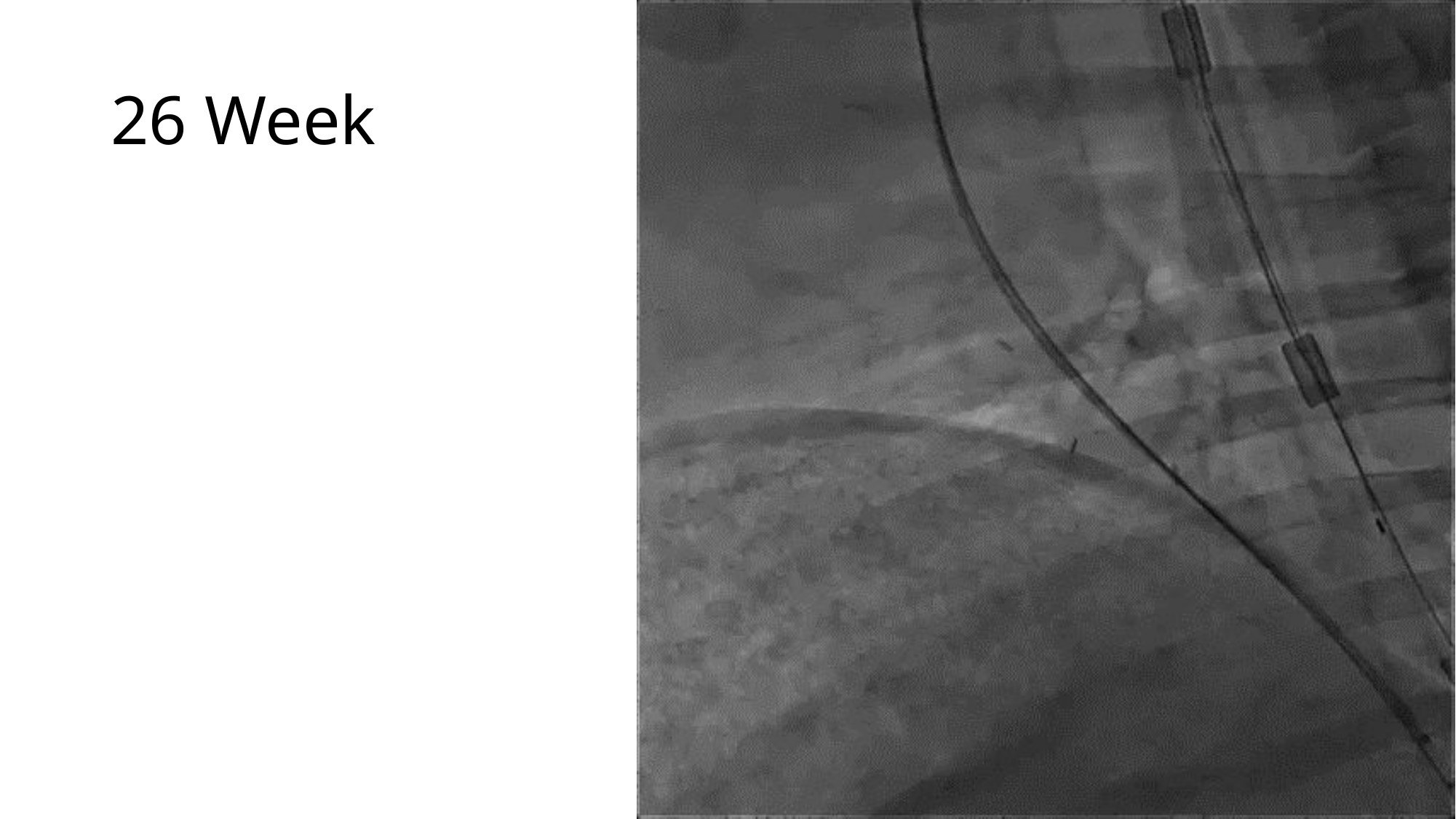

# 26 Week

## Slide 5
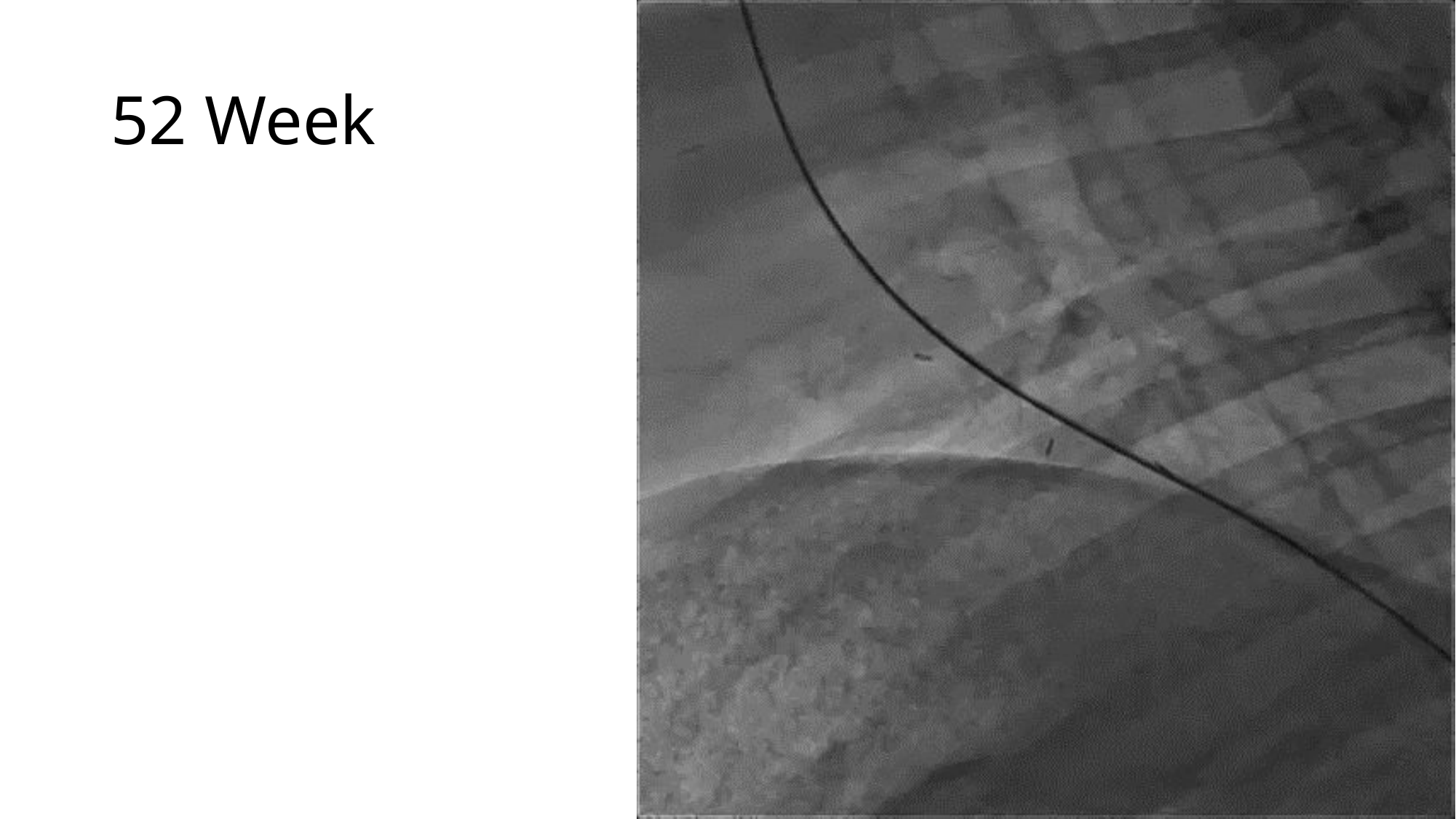

# 52 Week

## Slide 6
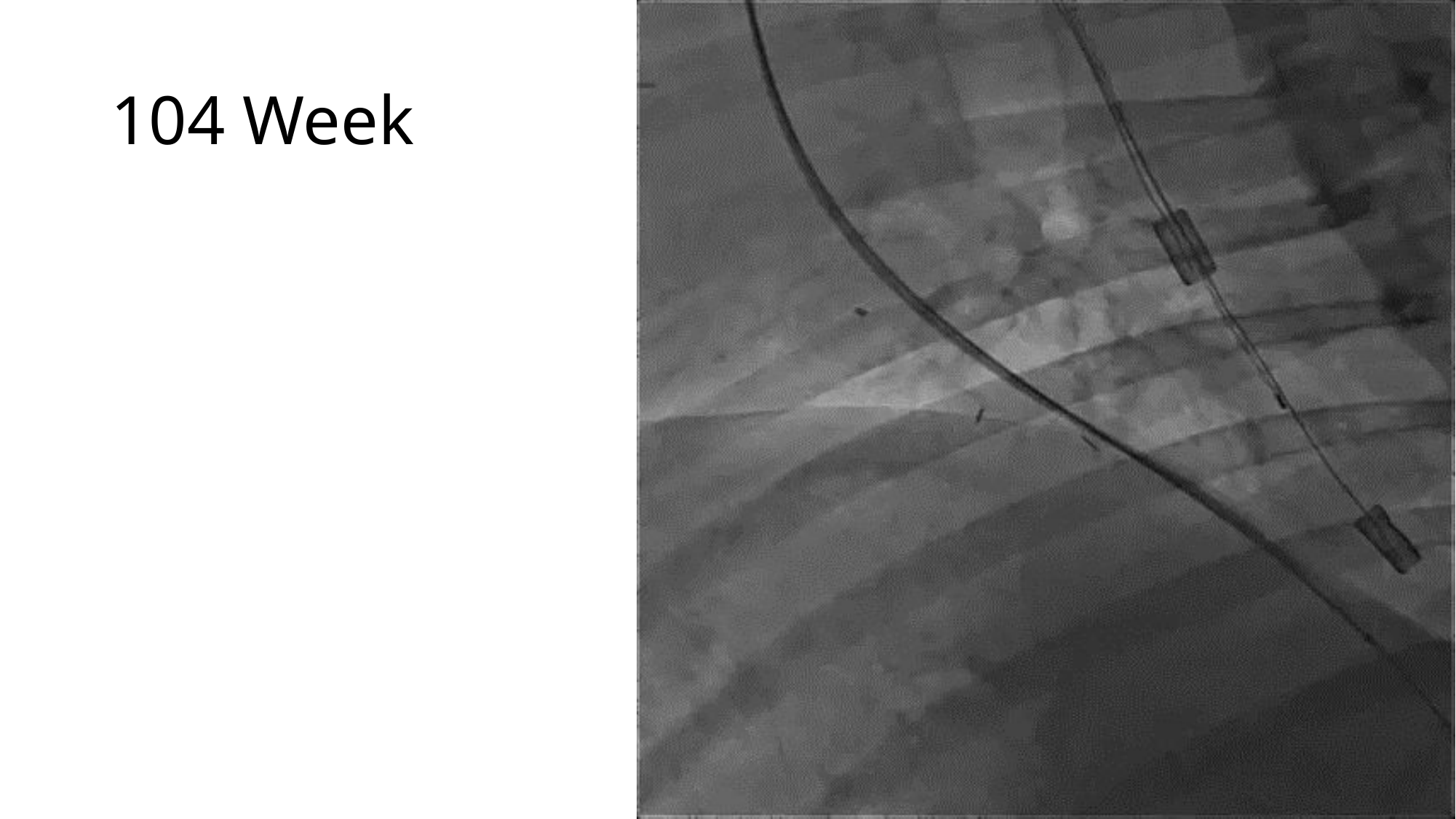

# 104 Week
