# Supplementary material for: Tissue engineered vascular grafts transform into autologous neovessels capable of native function and growth
Source: Commun Med (Lond). 2022 Jan 10;2:3. doi: 10.1038/s43856-021-00063-7 (PMC9053249; doi:10.1038/s43856-021-00063-7)
Supplement: Supplementary file 5 — Supplementary Video 2 [file 43856_2021_63_MOESM5_ESM.pptx]

## Slide 1
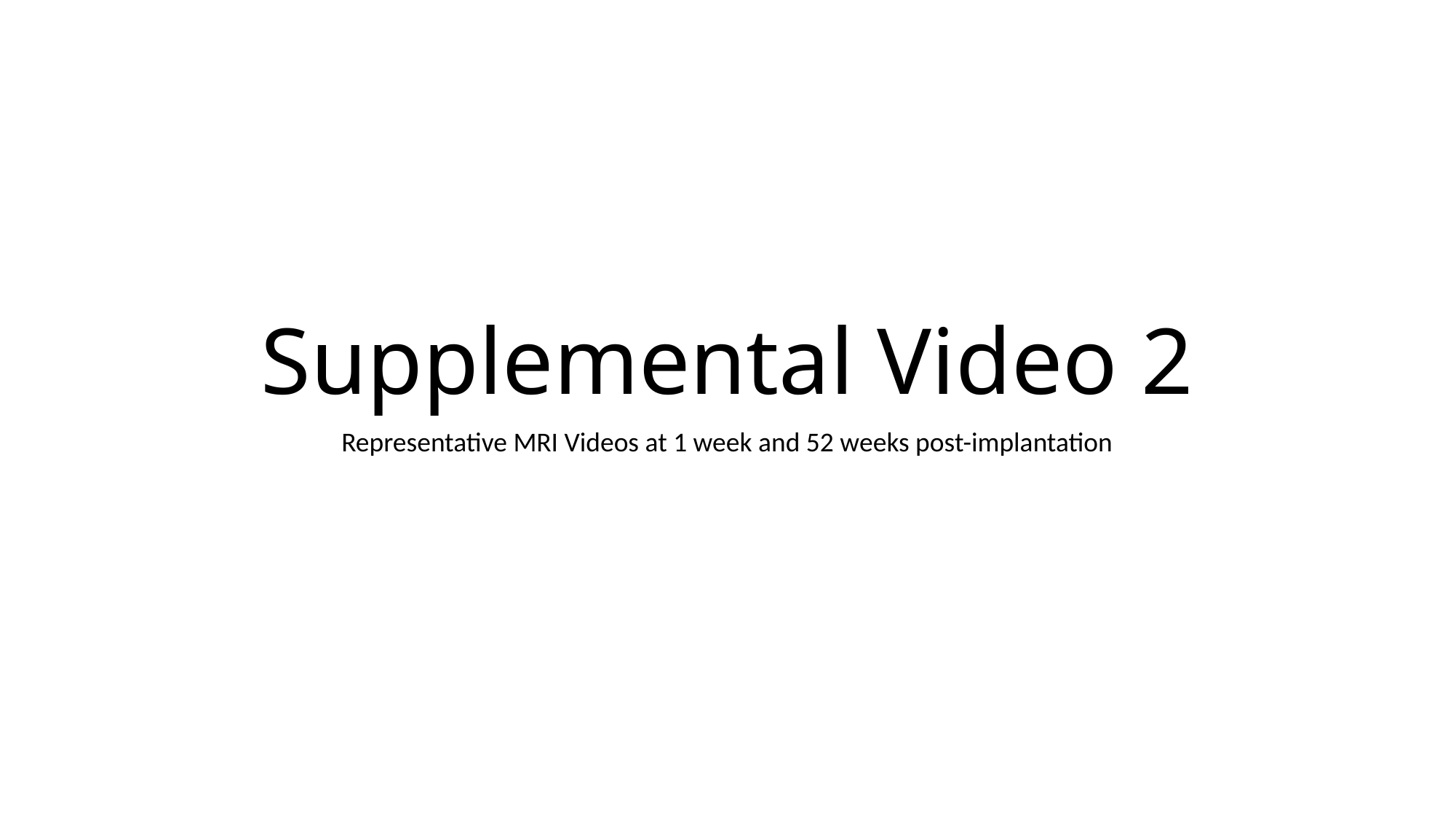

# Supplemental Video 2
Representative MRI Videos at 1 week and 52 weeks post-implantation

## Slide 2
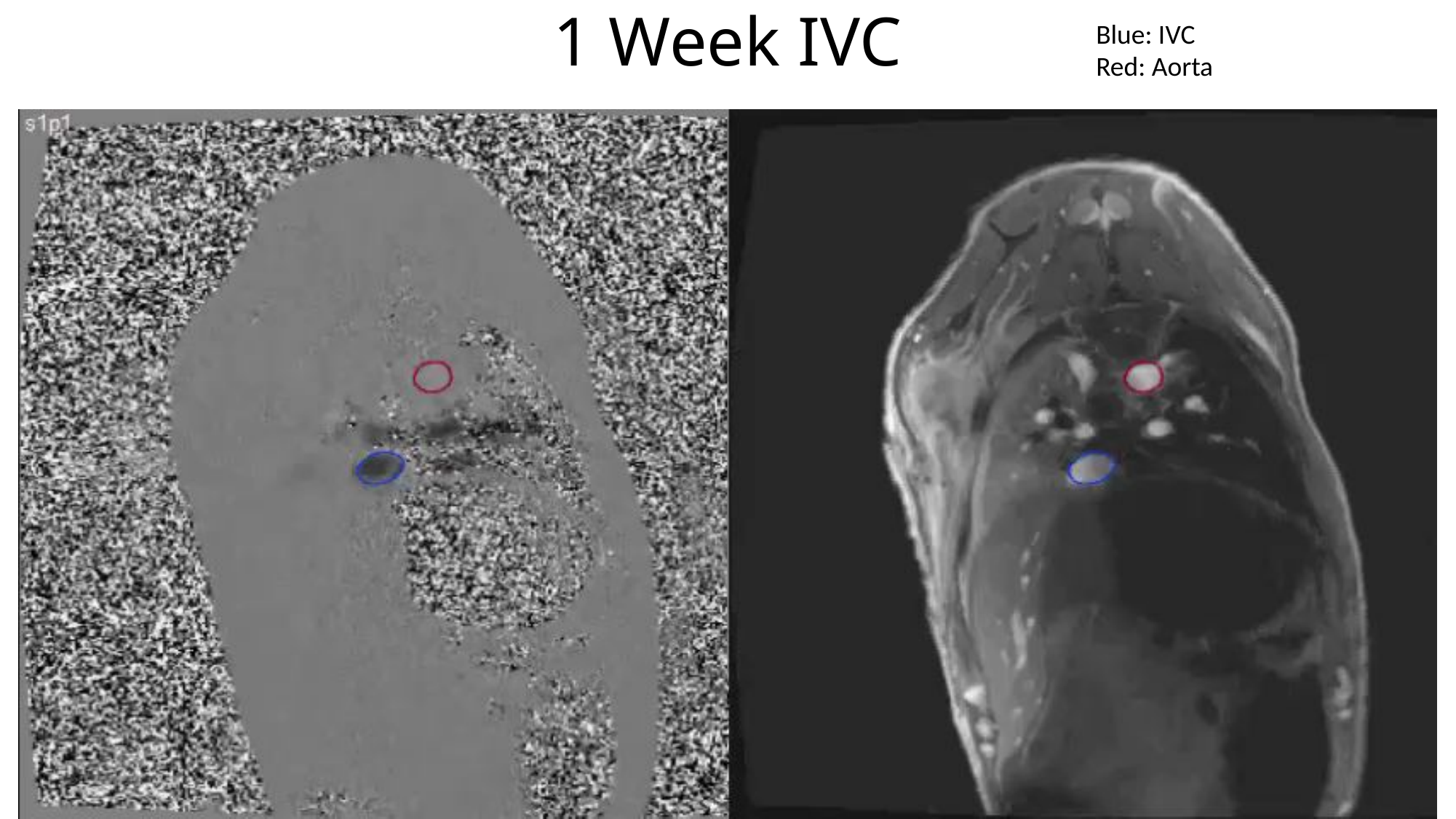

1 Week IVC
Blue: IVC
Red: Aorta

## Slide 3
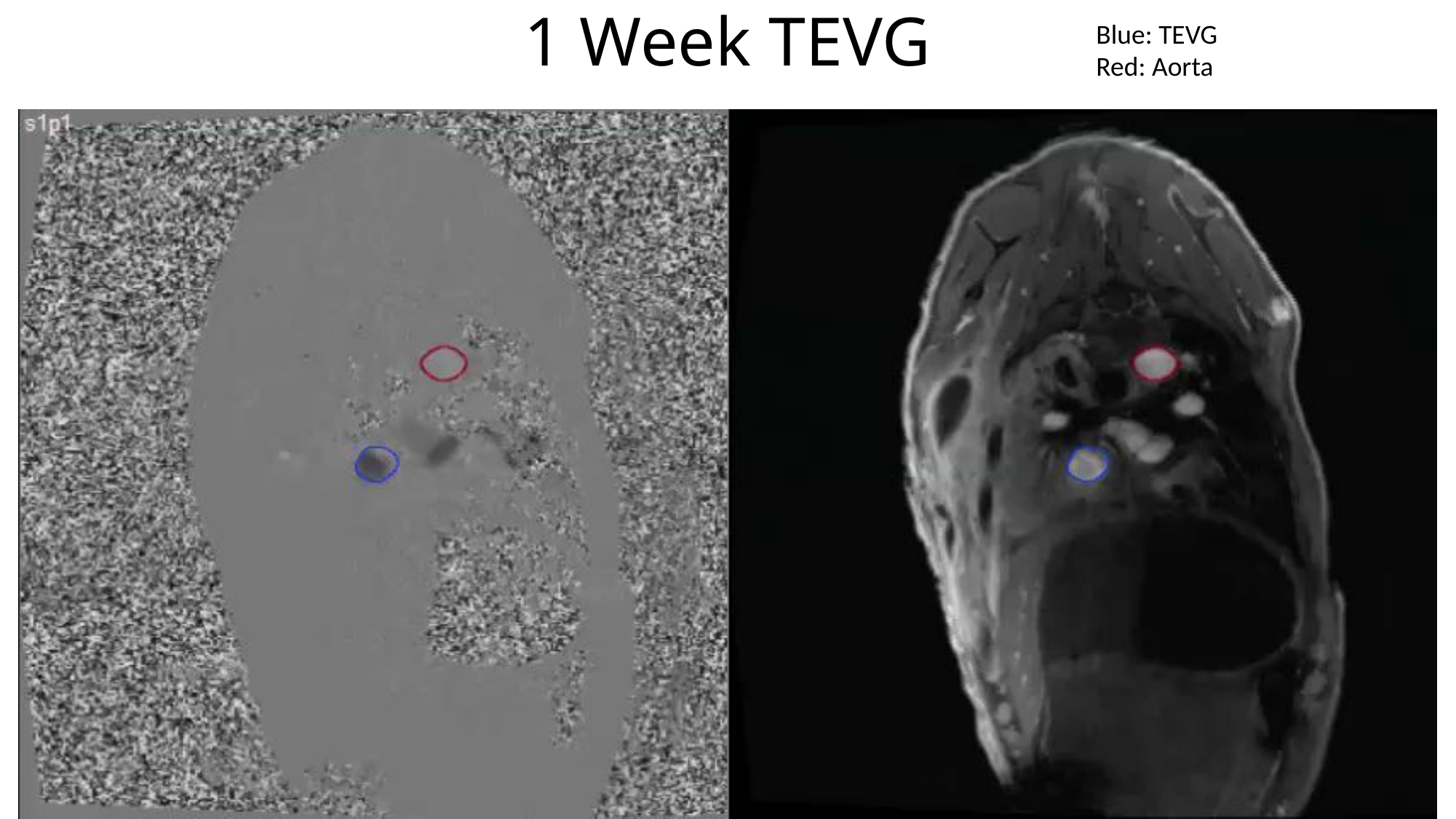

1 Week TEVG
Blue: TEVG
Red: Aorta

## Slide 4
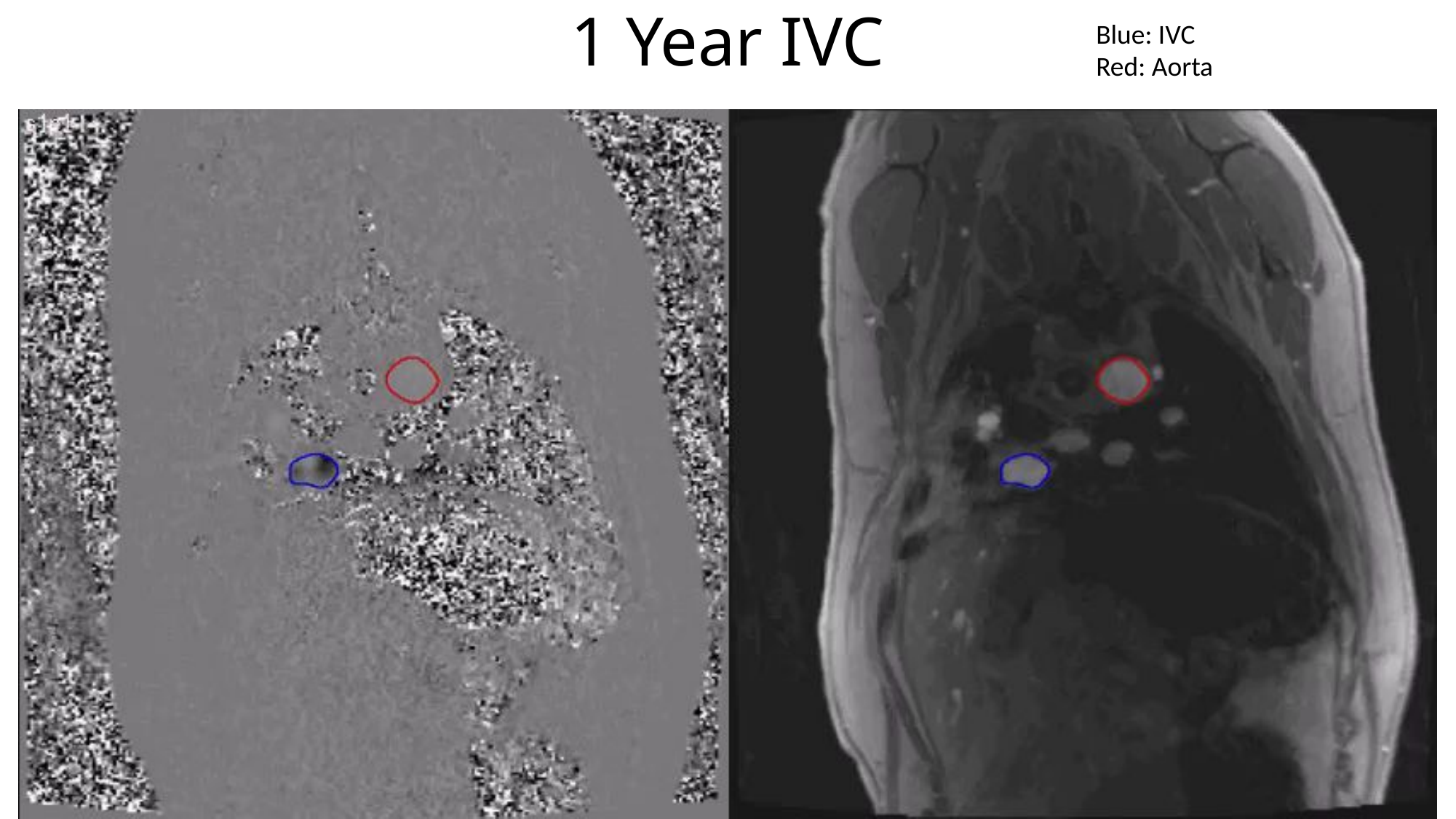

1 Year IVC
Blue: IVC
Red: Aorta

## Slide 5
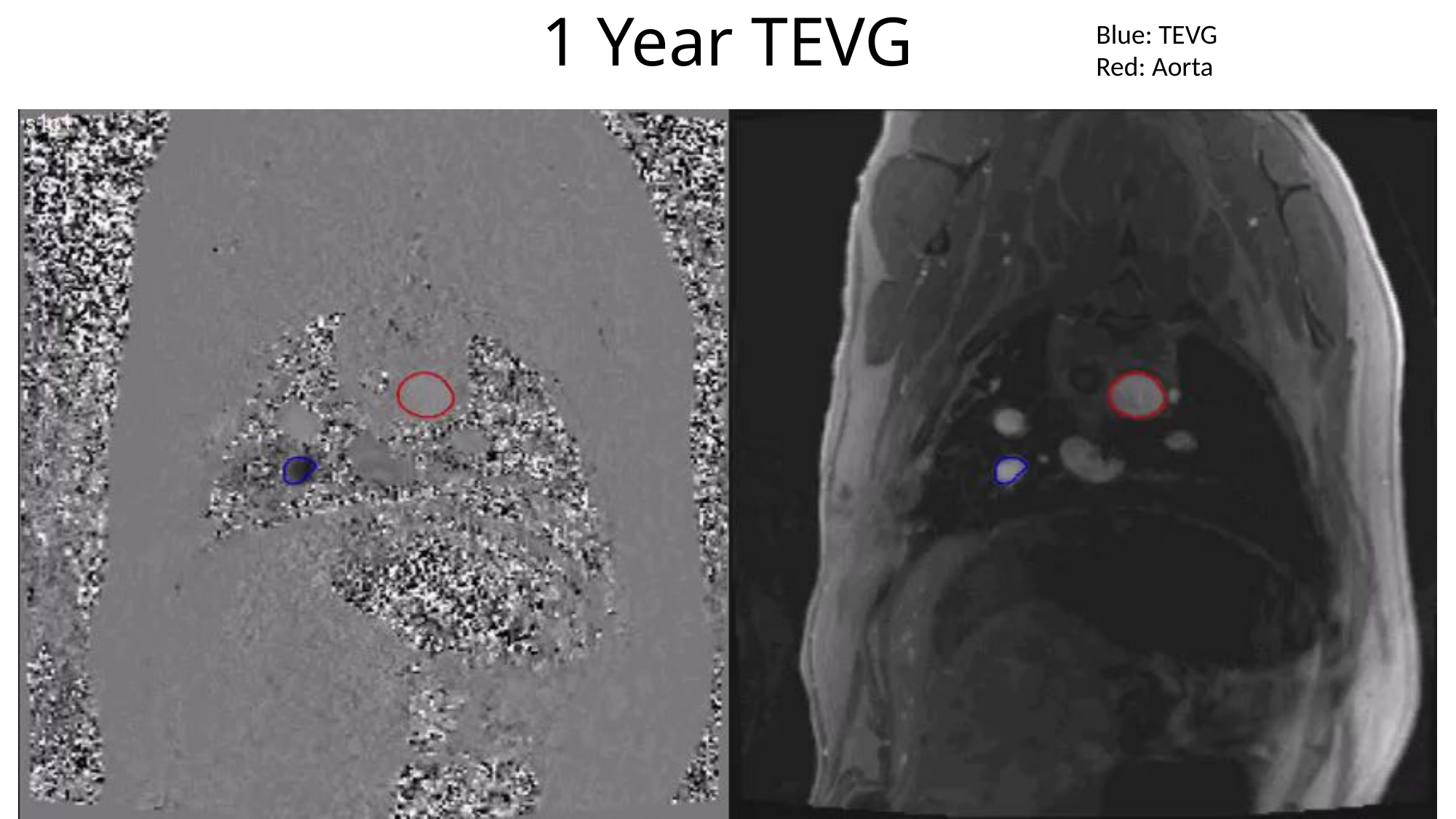

# 1 Year TEVG
Blue: TEVG
Red: Aorta
