# Supplementary material for: Tissue engineered vascular grafts transform into autologous neovessels capable of native function and growth
Source: Commun Med (Lond). 2022 Jan 10;2:3. doi: 10.1038/s43856-021-00063-7 (PMC9053249; doi:10.1038/s43856-021-00063-7)
Supplement: Supplementary file 7 — Description of Additional Supplementary Files [file 43856_2021_63_MOESM7_ESM.pdf]

Description of Additional Supplementary Files

File Name: Supplementary Data 1

Description: Raw Data used to create main figures in manuscript

File Name: Supplementary Data 2

Description: Description of animals utilized in each experiment

File Name: Supplementary Video 1

Description: Angiography. Representative angiography videos of implanted TEVGS at 1, 6, 26, 52, and 104 weeks post-implantation.

File Name: Supplementary Video 2

Description: MRI. Representative MRI videos of implanted TEVGs at 1 and 52 weeks postimplantation.
